# Exploring Novel Therapeutic Opportunities for Glioblastoma Using Patient-Derived Cell Cultures

**DOI:** 10.3390/cancers15051562

**Published:** 2023-03-02

**Authors:** Iwona A. Ciechomska, Kamil Wojnicki, Bartosz Wojtas, Paulina Szadkowska, Katarzyna Poleszak, Beata Kaza, Kinga Jaskula, Wiktoria Dawidczyk, Ryszard Czepko, Mariusz Banach, Bartosz Czapski, Pawel Nauman, Katarzyna Kotulska, Wieslawa Grajkowska, Marcin Roszkowski, Tomasz Czernicki, Andrzej Marchel, Bozena Kaminska

**Affiliations:** 1 Laboratory of Molecular Neurobiology, Nencki Institute of Experimental Biology, Polish Academy of Sciences, 02-093 Warsaw, Poland; 2 Postgraduate School of Molecular Medicine, Medical University of Warsaw, 02-091 Warsaw, Poland; 3 Department of Neurosurgery, Scanmed S.A. St. Raphael Hospital, 30-693 Cracow, Poland; 4 Institute of Psychiatry and Neurology, 02-957 Warsaw, Poland; 5 Department of Pathology, The Children’s Memorial Health Institute, 04-736 Warsaw, Poland; 6 Neurosurgery Department and Clinic, Medical University of Warsaw, 02-091 Warsaw, Poland

**Keywords:** glioblastoma, cancer stem cells, EMT, MGMT, temozolomide, doxorubicin, STAT3, EGFR inhibitor (AG1478)

## Abstract

**Simple Summary:**

Glioblastomas (GBM) are aggressive brain tumors with poor prognosis that need effective treatment. GBMs are characterized by extensive cellular and molecular heterogeneity which are reflected in patient-derived cell cultures, frequently used in testing potential therapeutics. Here, we established GBM-derived cell cultures from fresh tumor specimens and characterized them at the protein and molecular levels. We confirmed the considerable intertumor heterogeneity of GBMs. As the epidermal growth factor receptor (EGFR) is a subject of common oncogenic alterations in GBM, we tested anti-EGFR therapy combined with temozolomide (first-choice medication for GBM) or with doxorubicin (common therapeutic for various solid and blood cancers). We found that GBM-derived cells were more sensitive to a combined therapy than to monotherapy, particularly cells with inactive DNA repair mechanisms.

**Abstract:**

Glioblastomas (GBM) are the most common, primary brain tumors in adults. Despite advances in neurosurgery and radio- and chemotherapy, the median survival of GBM patients is 15 months. Recent large-scale genomic, transcriptomic and epigenetic analyses have shown the cellular and molecular heterogeneity of GBMs, which hampers the outcomes of standard therapies. We have established 13 GBM-derived cell cultures from fresh tumor specimens and characterized them molecularly using RNA-seq, immunoblotting and immunocytochemistry. Evaluation of proneural (OLIG2, IDH1^R132H^, TP53 and PDGFRα), classical (EGFR) and mesenchymal markers (CHI3L1/YKL40, CD44 and phospho-STAT3), and the expression of pluripotency (SOX2, OLIG2, NESTIN) and differentiation (GFAP, MAP2, β-Tubulin III) markers revealed the striking intertumor heterogeneity of primary GBM cell cultures. Upregulated expression of VIMENTIN, N-CADHERIN and CD44 at the mRNA/protein levels suggested increased epithelial-to-mesenchymal transition (EMT) in most studied cell cultures. The effects of temozolomide (TMZ) or doxorubicin (DOX) were tested in three GBM-derived cell cultures with different methylation status of the *MGMT* promoter. Amongst TMZ- or DOX-treated cultures, the strongest accumulation of the apoptotic markers caspase 7 and PARP were found in WG4 cells with methylated *MGMT*, suggesting that its methylation status predicts vulnerability to both drugs. As many GBM-derived cells showed high EGFR levels, we tested the effects of AG1478, an EGFR inhibitor, on downstream signaling pathways. AG1478 caused decreased levels of phospho-STAT3, and thus inhibition of active STAT3 augmented antitumor effects of DOX and TMZ in cells with methylated and intermediate status of *MGMT*. Altogether, our findings show that GBM-derived cell cultures mimic the considerable tumor heterogeneity, and that identifying patient-specific signaling vulnerabilities can assist in overcoming therapy resistance, by providing personalized combinatorial treatment recommendations.

## 1. Introduction

Glioblastoma (GBM) is a primary brain tumor, known to be one of the most aggressive human tumors. Despite maximal safe resection followed by radiation with adjuvant chemotherapy, the average survival of patients is 15 months after diagnosis and tumors recur within 6 months after therapy [1]. Temozolomide (TMZ) is an alkylating drug widely used as a first-choice chemotherapeutic agent in GBM [2], however, 50% of patients develop resistance to TMZ, which limits therapy outcomes. O^6^-methylguanine-DNA methyltransferase (MGMT) is responsible for removing the methyl group from O^6^-methylguanine in DNA, thereby diminishing the overall efficacy of TMZ. The expression of *MGMT* negatively correlates with promoter methylation and correlates with prolonged survival of GBM patients. In contrast, tumors with unmethylated *MGMT* (MGMT active) exhibit resistance to TMZ [3]. *MGMT* methylation allows for predicting TMZ effectiveness [4]. Additional molecular mechanisms may contribute to TMZ resistance, such as other DNA repair systems, epigenetic modifications, aberrant signaling pathways or molecular- and cellular heterogeneity in malignant glioma [5,6]. Therefore, there is an urgent need to discover a novel approach to increase glioma cell sensitivity to TMZ and other drugs.

Anthracycline antibiotic doxorubicin (DOX) is widely used in the treatment of various solid and blood cancers [7]. DOX is cytotoxic towards cultured glioma cells [8] and in animal models of malignant gliomas [9,10]. Unfortunately, DOX has low of blood-brain barrier (BBB) penetration, and causes side effects in healthy tissues, including dose-limiting cardiotoxicity. Various formulations such as nanoparticles, liposomes, exosomes and polymer conjugates were developed to improve transport of DOX through the BBB and achieve the desired drug concentration within tumors [11,12]. Complementary approaches such as combinatory treatment and/or intratumoral delivery of DOX have been used in GBM therapy to reduce side effects [11,13,14].

GBM is characterized by high inter- and intrapatient heterogeneity. Integrated genomic and transcriptomic analyses identified clinically relevant major subtypes of GBMs: classical (CL), mesenchymal (MES) and proneural (PN) [15]. These subtypes are tightly associated with genomic abnormalities. Platelet-derived growth factor receptor alpha (*PDGFRA*) amplifications and mutations in genes coding for isocitrate dehydrogenase 1 (*IDH1*) and tumor protein 53 (*TP53*) were most frequently found in the PN group. Epithelial growth factor receptor (EGFR) alterations were found in the CL group, while neurofibromin 1 (*NF1*) gene mutations occur preferentially in MES GBMs. Moreover, the therapy provides the greatest benefits in the CL-GBMs, and less/no benefits in the PN-GBMs [16]. Multi-region tumor sampling has shown co-existence of multiple subtypes in different regions of the same tumor. These subtypes can change over time and through therapy. Single-cell RNA-sequencing (scRNA-seq) indicated that distinct cells in the same tumor recapitulate programs from distinct subtypes [15,17,18]. Studies by Patel et al. [18] showed that cells from the same tumor had variable ‘stemness’ and expressed different receptor tyrosine kinases (RTKs). Markedly, several studies indicated the presence of different cells, including glioma stem cells (GSCs) (also called tumor-initiating cells), within a tumor and their contribution to tumor growth, recurrence and resistance to radio- and chemotherapies [19,20,21].

The amplifications and mutations of *EGFR* are detected in about half of GBM tumors and in 95% of CL-GBMs [22,23]. Amplification of *EGFR* is often accompanied by the appearance of an EGFR variant III (EGFRvIII), which lacks the extracellular domain, causing a constitutive ligand-independent activity [24]. EGFR and its downstream signaling networks contribute to GBM cell proliferation and diffused invasion [25]. Many EGFR-targeting therapies are in development or in clinical trials of many tumors, including GBMs [26,27]. Although EGFR kinase inhibitors had shown promising results in some tumors (e.g., non-small lung cancer), gefitinib and erlotinib had insignificant outcome in clinical trials [28,29]. Among mechanisms of therapy resistance to EGFR inhibitors are: *PTEN* (phosphatase and tensin homolog) alterations, deregulated PI3K (phosphatidylinositol 3-kinase) pathway [29,30], compensatory signaling pathways, tumor heterogeneity and ineffective BBB penetration [31].

One of the signaling pathways downstream EGFR involves a signal transducer and an activator of transcription 3 (STAT3), an oncogenic transcription factor [32] regulating the transcription of several genes involved in cell cycle progression, resistance to apoptosis, angiogenesis, invasiveness and immune escape [33,34]. GBM patients with high levels of activated (phosphorylated) STAT3 have more aggressive disease and poorer clinical outcomes [35]. Targeting STAT3 sensitizes glioma cells to anti-EGFR (Iressa/gefitinib) and alkylating agents [36]. Concurrent inhibition of EGFR and JAK2/STAT3 was highly effective in a panel of molecularly heterogeneous glioma stem cells (GSC) and in orthotopic *EGFRvIII* GSC xenografts [37]. Afatinib (a second generation of EGFR-inhibitor) combined with TMZ synergistically inhibited cell proliferation, clonogenicity, invasion and motility of cultured glioma cells expressing *EGFRvIII* and prevented progression of intracranially implanted U87-MG *EGFRvIII* cells [38]. Cetuximab (an anti-EGFR antibody) augmented radiation and chemotherapy effects in GBM cells in vitro and in vivo [39,40]. TMZ and cetuximab were tested in a phase I/II clinical trial of primary GBMs [41]. Depatuxizumab mafodotin (ABT-414), an EGFR-targeting antibody–drug conjugate [42], selectively killed tumor cells overexpressing wild-type or mutant forms of EGFR and reduced glioma growth in mice [43].

DOX conjugated with ultrasmall nanoparticles showed a significant efficacy in patient-derived xenografts harboring EGFR mutations and/or amplification after intravenous administration [14]. The anti-EGFR-doxorubicin-loaded immunoliposomes (ILs-DOX) displayed highly efficient binding and internalization in a panel of *EGFR* and EGFRvIII overexpressing cells [44,45]. A small trial with anti-EGFR ILs-DOX on relapsed GBMs with *EGFR* amplification showed positive response in one patient [46].

The lack of effective conventional GBM therapy encourages researchers to search for new therapeutic strategies based upon the combination or repurposing of drugs. We tested the effects of an EGFR inhibitor AG1478 combined with TMZ or DOX using molecularly diverse patient-derived cell cultures, especially with a different status of *MGMT*. We established a quick and reliable method for generating patient-derived primary glioma cell cultures and performed their molecular characterization. We demonstrate that blocking EGFR signaling together with TMZ or DOX decreased cell viability and induced apoptosis of GBM-derived cells with no or low expression of *MGMT*. Mechanistic studies showed that although AG1478 inhibits phosphorylation of STAT3 in patient-derived cells, it was not sufficient to sensitize primary cells with the unmethylated *MGMT* promoter. The data define cell-type specific responses to the EGFR inhibitor in combination with TMZ or DOX and highlight a role of the *MGMT* promoter methylation in predicting cell responses to chemotherapeutics.

## 2. Materials and Methods

### 2.1. Cell Cultures and Treatments

WG0, WG1, WG3, WG4, WG5, WG6, WG9, WG10, WG13, WG14, WG15, WG16, WG16, WG17, WG18, WG19 primary glioma cultures originated from surgically resected glioblastoma samples (grade 4, according to WHO 2016 classification) [47]. The use of tissues was approved by the Research Ethics Board at Institute of Psychiatry and Neurology in Warsaw, Poland, and informed consents were obtained from the patients. All methods were carried out in accordance with the relevant guidelines and regulations. Freshly resected tumor tissues were washed in Hank’s balanced sodium solution (HBSS; Gibco Life Technologies, Rockville, MD, USA) and subjected to mechanical and enzymatic dissociation using a Neural Tissue Dissociation Kit (Miltenyi Biotec, Bergisch Glasbach, Germany) according to the manufacturer’s instructions. Some samples were processed without enzymatic digestion, in favor of accurate tissue cutting in DMEM/F12 medium until a smooth, milky single-cell suspension was achieved. To remove undissociated pieces and debris, the cell suspension was filtered through 100- and 40-micron cell strainers. The blood cells were discarded during serial passage and medium exchange, instead of using Lympholyte-M [48]. Tumor cells were resuspended in DMEM/F-12 medium (Gibco Life Technologies, Rockville, MD, USA) supplemented with 10% fetal bovine serum for adherent cultures, or DMEM/F-12 serum-free medium for sphere cultures, and plated at a density of 1–2 × 10^6^ cells/T75 flask. Every 4 days, 50% of the fresh medium was replaced. 

The L0125 and L0627 GBM GSC lines were provided by Dr Rossella Galli (San Raffaele Scientific Institute, Milan, Italy) [49]. L0125 and L0627 were expanded in vitro in serum-free medium for sphere culture.

Normal human astrocytes (NHA) were purchased from Lonza (Walkersville, MD, USA) and cultured in ABM Basal Medium (Lonza) supplemented with 3% fetal bovine serum, 1% L-glutamine, 0.1% ascorbic acid, 0.1% human EGF, 0.1% gentamicin, and 0.0025% recombinant human insulin. 

NTERA-2 cl.D1 were purchased from ATCC (Manassas, VA, USA) and cultured in DMEM with GlutaMax-1 and supplemented with 10% fetal bovine serum.

All cell cultures were grown in a humidified atmosphere of CO_2_/air (5%/95%) at 37 °C.

### 2.2. Sphere Cultures and Material Collection

For sphere cultures, cells were seeded at a low density (3000 viable cells/cm^2^) onto non-adherent plates and cultured in serum-free DMEM/F-12 medium, supplemented with 2% B27 (Gibco Life Technologies, Rockville, MD, USA), 20 ng/mL recombinant human bFGF (Miltenyi Biotec, Bergisch Gladbach, Germany), 20 ng/mL recombinant human EGF (StemCell Technologies, Vancouver, BC, Canada), 0.0002% heparin (StemCell Technologies, Vancouver, BC, Canada) and antibiotics (100 U/mL penicillin, 100 µg/mL streptomycin, Gibco Life Technologies, Rockville, MD, USA). Every 3 days, 25% of the medium was replaced. After 7–14 days of culturing, the spheres were collected by centrifugation at 110× *g* and lysed in Qiagen RLT lysis buffer for RNA isolation or lysed in buffer supplemented with complete protease inhibitor cocktail (Roche Applied Science, Indianapolis, IN, USA) for blotting.

### 2.3. Cell Treatments

Temozolomide (TMZ), doxorubicin (DOX) and AG1478 (AG) were dissolved in DMSO. Cells were treated with single drugs: TMZ (1 mM) for 72 h, DOX (50–1000 nM) for 48 h and AG (10 µM) for 6 h, or with a combination of TMZ + AG (1 mM + 10 µM) for 72 h, and DOX + AG (500 nM + 10 µM) for 48 h. DMSO was added at respective concentrations and served as a control condition.

### 2.4. Cell Viability Assays

Cell viability was evaluated using the MTT metabolism test, as described previously [50]. Briefly, 1.5–2 × 10^4^ cells were seeded onto 24-well plates and the MTT solution (0.5 mg/mL; Sigma-Aldrich, Taufkirchen, Germany) was added 24, 48, 72 and 96 h after cell seeding. After 1 h of incubation at 37 °C, water-insoluble formazan was dissolved in DMSO and optical densities were measured at 570 nm and 620 nm using a scanning multi-well spectrophotometer. Cell viability after AG, DOX or TMZ treatments was evaluated using the PrestoBlue Cell Viability Reagent (Invitrogen, Eugene, OR, USA). Diluted PrestoBlue reagent was added to each well for 1.5 h at 37 °C. After collecting samples, fluorescence was measured at 570 nm and 620 nm using a multi-well spectrophotometer.

### 2.5. Immunoblotting

Whole cell lysates were prepared in a buffer containing phosphatase and protease inhibitors, separated by SDS-PAGE and transferred onto nitrocellulose membranes as described [51]. After blocking with 5% nonfat milk in a blocking buffer, the membranes were incubated overnight with primary antibodies and then with the appropriate secondary antibodies for 1 h. Immunocomplexes were visualized using an enhanced chemiluminescence detection system (SuperSignal West Pico PLUS; ThermoFisher Scientific, Rockford, IL, USA). Blots were visualized with a Chemidoc imaging system (Bio-Rad, Hercules, CA, USA). The molecular weight of proteins was estimated with prestained protein markers (Sigma-Aldrich, St. Louis, MO, USA). Densitometric analysis of the blots and quantification of the results from independent experiments were performed, and the levels of a protein of interest were compared to its levels in NHA, taken as 1, and marked by a solid black line. 

### 2.6. Immunofluorescence

Cells were seeded onto a glass coverslip at a density of 2–3 × 10^4^ cells. After 24 h, cells were fixed with 4% PFA at pH 7.2, washed, permeabilized with 0.1% Triton-X100 and blocked in a mix of 2% donkey serum and 1.5% fetal bovine serum, followed by overnight incubation with primary antibodies diluted in PBS containing 1% bovine serum albumin (BSA) and 0.1% Triton X-100. Cells were then washed in PBS, incubated with Alexa Fluor A555 secondary antibodies diluted in PBS for 2 h, counterstained with DAPI and mounted. For reagent specifications, catalogue numbers, and concentrations, see Supplementary Table S1.

### 2.7. Scratch-Wound Assay

Cells were seeded onto 60-mm culture dishes at a density of 8 × 10^4^ cells, in duplicates. When cells reached 80% confluency, a scratch was gently made using a p200 pipette tip. Pictures of the area were taken immediately after a wound was inflicted to the cells (0 h) and after 18 h. The migration rate was estimated from the distance that the cells moved, as determined microscopically. The area between the edges of the wound was measured by using Image J software. Six measurements were taken for each experimental condition. A mobility rate is expressed as percentage of wound closure as compared to 0 h time point. Migration rates were calculated using the following equation: (initial distance − final distance/initial distance) × 100%.

### 2.8. Bisulfite DNA Conversion and Methylation-Specific Polymerase Chain Reaction (MS-PCR)

DNA was extracted using standard phenol/chloroform methods. The purity and concentration of the DNA were estimated by measuring absorbance at 260/280 nm. DNA (2 μg) was treated with bisulfite (EpiTect Bisulfite Kit, Qiagen, Hilden, Germany). The modified DNA was amplified using primers specific for the methylated or unmethylated *MGMT* gene promoter, as listed in Supplementary Table S1. Each PCR mixture contained 1 μL of DNA, 500 nM of primers, 1 reaction buffer containing 1.5 mM MgCl_2_, and 1 U HotStarTaq DNA polymerase and 250 mM dNTPs (Promega, Madison, WI, USA). PCR was performed with thermal conditions as follows: 95 °C for 10 min, 45 cycles of 95 °C for 30 s, 57 °C for 30 s and 72 °C for 30 s, with a final extension of 72 °C for 10 min. PCR products were visualized using Agilent TapeStation system (Agilent Technologies, Santa Clara, CA, USA) yielding a band of 81 bp for a methylated product and 93 bp for an unmethylated product. Positive methylated and positive unmethylated controls (EpiTect PCR Control DNA Set Qiagen, Germany) were included.

### 2.9. Quantitative RT-PCR Analysis

Total RNA was extracted using an RNeasy Mini kit (Qiagen, Germany) and purified using RNeasy columns. The integrity of the RNA was determined using an Agilent 2100 Bioanalyzer. For qRT-PCR, the total RNA from cells was used to synthesize cDNA by extension of oligo (dT)_15_ primers with SuperScript reverse transcriptase (Thermo Fisher Scientific, USA). Real-time PCR experiments were performed in duplicates using a cDNA equivalent of 22.5 ng RNA in a 10 μL reaction volume containing 2x SYBR Green Fast PCR Master Mix (Applied Biosystems, Foster City, CA, USA) and a set of primers. Sequences of the primers are listed in Table S1. Data were analyzed by the relative quantification method using StepOne Software (Applied Biosystems, Foster City, CA, USA). The expression of each product was normalized to 18S rRNA and presented as a dCt value.

### 2.10. mRNA Library Preparation and Sequencing

The quality and quantity of isolated nucleic acids were determined by Nanodrop (Thermo Fisher Scientific, Waltham, MA, USA). mRNA libraries were prepared using a KAPA Stranded mRNA-seq Kit (Kapa Biosystems, Cape Town, South Africa) according to manufacturer’s protocol. Briefly, mRNAs were enriched from 500 ng total RNAs using poly-T oligo-attached magnetic beads (Kapa Biosystems). Enriched mRNA was fragmented, and then the first and second strands of cDNA were synthesized. Adapters were ligated and the loop structure of each adapter was cut by a USER enzyme (NEB, Ipswich, MA, USA). Finally, the amplification of obtained dsDNA fragments that contained a specific adapter sequence was performed using NEB starters. Quality control of the final libraries was performed using an Agilent Bioanalyzer High Sensitivity dsDNA Kit (Agilent Technologies, Waldbronn, Germany). The concentration of the final libraries was measured using the Quantus Fluorometer and QuantiFluor ONE Double Stranded DNA System (Promega, Madison, WI, USA). Libraries were sequenced on a HiSeq 1500 (Illumina, San Diego, CA, USA) on the rapid run flow cell with a paired-end setting (2 × 76 bp).

### 2.11. RNA-seq Data Alignment, Processing and Analysis

Data analysis: RNA sequencing reads were aligned to the human genome reference with the STAR algorithm [52], a fast gap-aware mapper. Then, gene counts were obtained by featurecounts [53] using human transcriptomic annotations. The counts were then imported to R and processed by DESeq2 [54]. The counts were normalized for gene length and library size.

TCGA public data analysis: TCGA level 3 RNA-seq data (aligned by STAR and gene expression counted by HTseq) were uploaded to R. Data from TCGA GBM (glioblastoma, WHO grade 4) and LGG (lower-grade gliomas, WHO grades 2/3) repositories were uploaded. Gene expression values as FPKM (fragments per kilobase of exon per million) were used for further analysis. The curated sets of genes characteristic for each GBM subtype, categorized originally by Verhaak et al. [16], were downloaded from the Molecular Signatures Database v7.5.1. The analysis for the following gene sets was performed: VERHAAK_GLIOBLASTOMA_PRONEURAL, VERHAAK_GLIOBLASTOMA_NEURAL, VERHAAK_GLIOBLASTOMA_CLASSICAL, VERHAAK_GLIOBLASTOMA_MESENCHYMAL.

### 2.12. Statistical Analysis

All biological experiments were performed on 3–4 independent cell passages. Results were expressed as means ± standard deviation (SD). *p*-values were calculated using a two-tailed *t*-test or a one-way ANOVA followed by an appropriate post hoc test using GraphPad Prism v6 (GraphPad Software, Boston, MA, USA). Differences were considered statistically significant for *p* values < 0.05.

The effect size, Cohen’s ‘*d*’ and Hedge’s ‘*g*’ [55] were calculated as follows: $d=\frac{\bar{x_{1}}-\bar{x_{2}}}{\sqrt{MS_{E}}}$, $g=d\times \left(1-\frac{3}{4\left(n_{1}+n_{2}\right)-9}\right)$, where $\bar{x}$ is the mean of the group, $MS_{E}$ is the error mean square, and n  is the sample size.

## 3. Results

### 3.1. Generation and Phenotypic Characterization of Patient-Derived Glioma Cell Cultures

**Figure 1:**
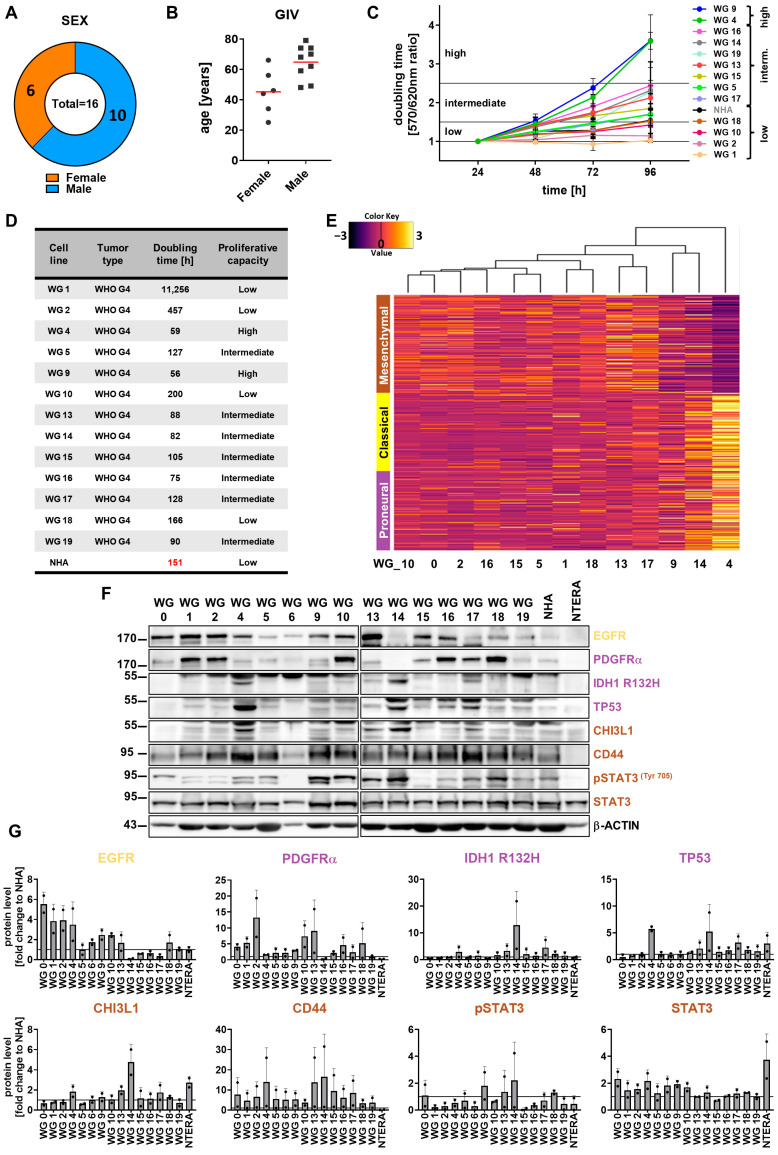
Characterization of glioma patient cohort and transcriptomic and protein-based subtyping of patient-derived primary cell cultures. (**A**,**B**) Graphical representation of sex (**A**) and age (**B**) of patients in the analyzed glioma cohort. Red line represents the mean age. (**C**) The proliferation capacity of glioblastoma-patient-derived primary cell cultures and normal human astrocytes (NHA) determined by MTT metabolism assay, *n* = 3, mean ± SD. (**D**) Doubling time of primary glioma cell cultures and NHA as a non-malignant control. (**E**) Transcriptomic data of patient-derived cell cultures represented as a heatmap with Verhaak signatures (MES, CL and PN) for glioblastoma subtypes. (**F**) Representative immunoblots illustrating the markers of glioblastoma subtypes: IDH1 ^R132H^, CHI3L1, pSTAT3, STAT3 (MES); EGFR (CL); and PDGFRα, TP53 (PN) in cultures of human glioma cells, NHA and NTERA-2 cells (NTERA). (**G**) Quantification of immunoblots. The level of a protein of interest was compared to the level of NHA. NHA cells were set equal to 1 and marked by a solid black line. β-actin was used as a loading control. NHA served as a non-malignant control, whereas NTERA served as a positive control for stemness properties, *n* = 2, mean ± SD. The whole blots and molecular weight markers are shown in Supplementary Figure S8.

Patient-derived cell cultures represent more reliable cellular models for testing new cytotoxic drugs in comparison with commercially established cell lines. Thus, we aimed to establish primary cell cultures from freshly resected high- or low-grade gliomas. Following dissociation of the tissue, cells were cultured in the presence of serum as adherent cells or as spheres in defined serum-free media. We succeeded in obtaining adherent cell cultures from the vast majority of glioma samples, whereas only two tumor samples gave rise to spheres, enriched in GSCs. Cell cultures with unchanged proliferation after eight subsequent passages were considered as glioma cell lines. Three cell cultures (WG0, WG3 and WG6) underwent two passages and stopped growing. Altogether, we established 13 GBM-derived cell lines out of 16 WHO high-grade glioma surgical specimens. We managed to develop two cell cultures from a WHO grade 1 tumor, and two cell lines from WHO grade 2 or 3 tumors. Information concerning age, sex and histopathological diagnosis of patients is presented in the table (Supplementary Figure S1). The mean age of men was 65, and 45 in the case of women (Figure 1B). Our studies have shown that GBMs were more frequent in men than in women in a 1.67:1 proportion (Figure 1A,B). Sex differences in GBM incidence have been previously reported [56]. 

We analyzed the doubling time of 13 patient-derived cell cultures (Figure 1C,D) within 4 days. Cells with a doubling time of less than 60 h were considered as highly proliferating cells, where as those dividing every 60–150 h were designated as intermediate-proliferating cells. Finally, cell lines with a doubling time of more than 150 h were marked as slowly proliferating cells. Only WG4 and WG9 cells had a high proliferative index. Most of the cells were intermediate-proliferating cells, with an average doubling time of 100 h. WG18, WG10, WG2 and WG1 were slowly dividing cells. There was no correlation between the doubling time of patient-derived cell cultures and patient age (Figure 1C,D; Supplementary Figure S1). The doubling time of normal human astrocytes (NHA), used as a non-malignant control, was approximately 150 h. The proliferation rate of commercial glioma cell lines U251, U87-MG, LN229 and LN18 were 57, 43, 41 and 40 h, respectively, indicating that established glioma cell lines grew much faster than primary glioma cell cultures. 

For better characterization of primary cell cultures, we applied the RNA-seq and the unsupervised analysis of mRNA expression profiles, which compared 13 GBM-derived primary cell cultures to the TCGA (The Cancer Genomic Atlas) glioma datasets [16]. The results were mapped using PCA (principal component analysis). The resulting PCA (data not shown) showed a clear separation of low- and high-grade gliomas in the TCGA dataset, and primary GBM-derived cell cultures were more similar to high-grade gliomas in terms of transcriptional profiles. Gliomas from TCGA and the primary glioma cell cultures clustered separately (data not shown). 

Next, we attempted to assign each individual primary GBM cell culture to four molecular subclasses. Most of the patient-derived cell cultures represented mixed subtypes, without a dominant gene expression signature, with the exception of WG13 and WG17 showing mostly the MES signature, and WG4 and WG14 represented by mixed, PN and CL subtypes (Figure 1E). Then we performed cell culture subtyping by assessing protein expression, as previously demonstrated [57,58]. We analyzed the levels of seven proteins by Western blotting (Figure 1F,G). Detection of mutant IDH1^R132H^ and high levels of TP53 and PDGFRα suggested the PN subtype, high levels of EGFR evidenced the CL subtype, and high levels of CHI3L1/YKL40, CD44 and phospho-STAT3 were typical for the MES subtype. We found that WG4, WG14 and WG17 were positive for the mutant IDH1^R132H^ and TP53, but had low expression of PDGFRα. High levels of EGFR were detected in approximately 50% of the tumor cell cultures (WG0, WG1, WG2, WG4, WG9, WG10, WG13, WG18). Immunoblot analysis with antibodies specifically recognizing mesenchymal markers demonstrated elevated CD44 levels in all tested glioma cell cultures, whereas high expression of CHI3L1/YKL40 was found only in WG4 and WG14 cells. Elevated levels of phosphorylated STAT3 (active form of STAT3) were observed in the WG9, WG10, WG13, WG14, and WG18 primary glioma cell lines. The global gene profiling and protein-based classification of primary glioblastoma cell cultures revealed that WG4 and WG14 could be classified into the PN subtype; however, exome sequencing of the WG4 cells did not detect any mutation in *IDH1* (data not shown).

### 3.2. Stem Cell Capacity of WG4 and WG14 Cells

**Figure 2:**
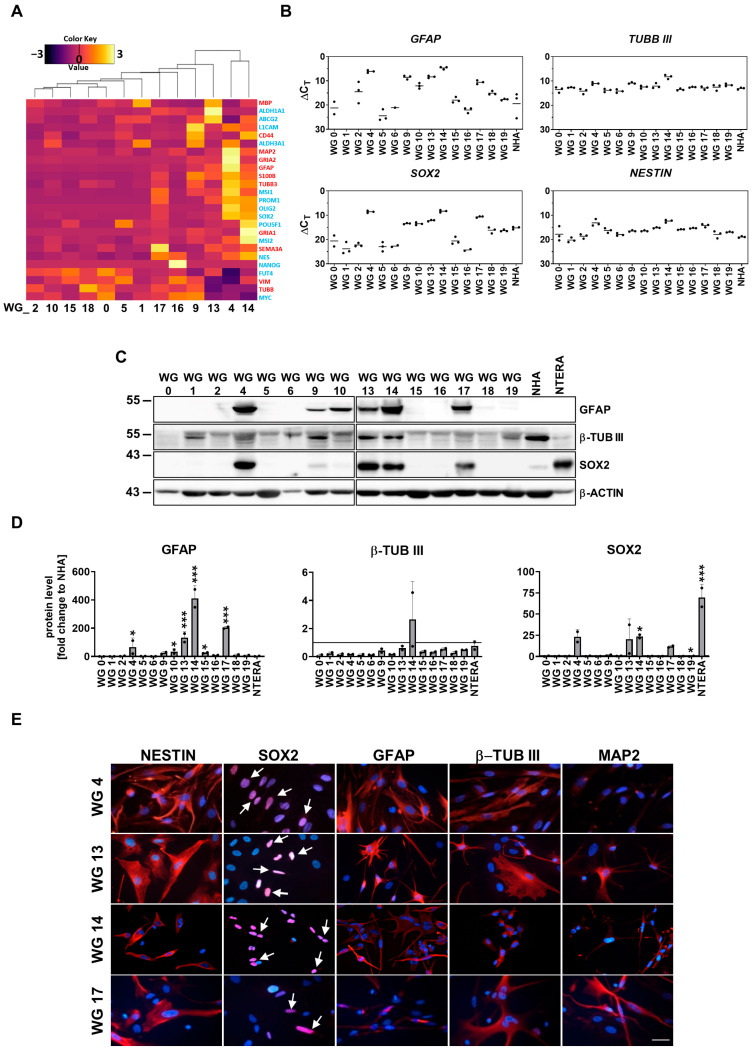
Stemness and differentiation markers across primary cell cultures. (**A**) RNA-seq of primary cell cultures represented as heatmap with stemness (indicated by blue color) and differentiation (red color) related genes. (**B**) Expression of selected differentiation (*GFAP, TUBBIII*) and stemness (*SOX2, NESTIN*) related genes in human patient-derived primary cultures and NHA as a control. The RT-qPCR data are shown as delta Ct values relative to the *18S* expression. (**C**) Representative immunoblots showing GFAP, β-TUB III and SOX2 levels in glioma primary cultures, NHA and NTERA cells. (**D**) Quantification of immunoblots. The level of a protein of interest in control cells equals 1 and is marked by a solid black line. β-actin was used as a loading control. NHA serves as a non-malignant control, whereas NTERA serves as a positive control with stemness properties. Statistical analysis was performed using one way ANOVA with Dunnett’s post hoc test to NHA cells (* *p* < 0.05, *** *p* < 0.001), *n* = 2, mean ± SD. (**E**) Representative immunofluorescent staining of WG4, WG13, WG14 and WG17 cells displaying the stemness properties, toward the stemness (NESTIN, SOX2) and differentiation (GFAP, β-TUB III, MAP2) markers. White arrows indicate positive nuclear staining. Scale bar: 100 µm. The whole blots and molecular weight markers are shown in Supplementary Figure S9.

GBMs contain a rare population of glioma stem-like cells (GSCs, also called glioma-initiating cells) with capacities for self-renewal, multi-lineage differentiation, and resistance to therapies [20]. The expression of selected pluripotency and differentiation markers was examined. Transcriptomic analysis revealed high expression of both types of markers in WG4 and WG14 cells in comparison with other primary cell cultures (Figure 2A). We found high expression of pluripotency markers such as *PROM1*, *OLIG2*, *SOX2* and *NESTIN* (a marker of neural precursors), as well as overexpression of markers for astrocytes (*GFAP*, *S100*) and neurons (*MAP2*, *TUBB3*). These results were validated by quantitative RT-PCR analysis, Western blotting and immunostaining (Figure 2B–E). High expression of GFAP at the mRNA and protein levels was observed not only in WG4 and WG14 but also in WG9, WG10, WG13 and WG17. Interestingly, these cell cultures exhibited high expression of *TUBB3, SOX2* and *NESTIN* mRNA. Elevated levels of GFAP, β-Tubulin III, SOX2 and NESTIN proteins were detected in WG9, WG13, and WG17 cells (Figure 2B–E, Supplementary Figure S2). Using Western blotting analysis and immunostaining, we did not detect the OLIG2 protein expression in all tested glioma cell lines growing in serum-containing media (Supplementary Figure S2). Overexpression of both pluripotency and differentiation markers was observed in the same cell lines and confirmed that the tumor cells are aberrantly differentiated. Moreover, these results revealed the inter- and intratumoral heterogeneity of GBM-derived cell cultures. 

**Figure 3:**
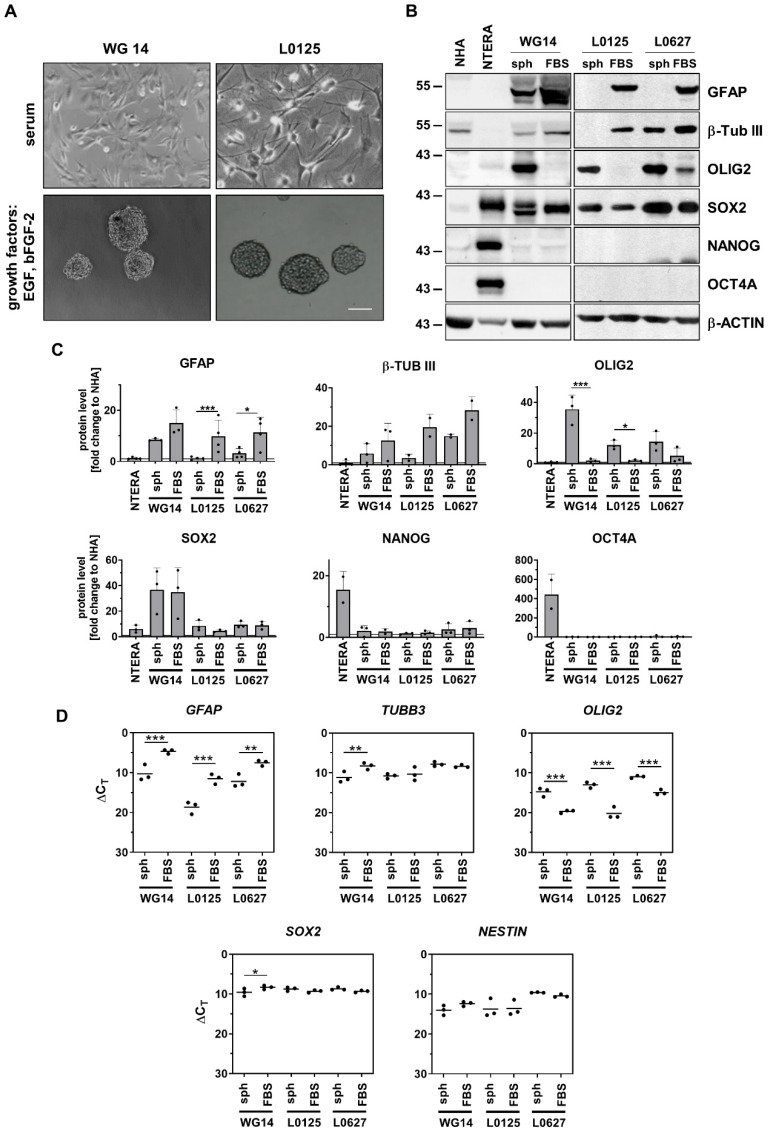
Characterization of stemness properties of GBM-derived sphere and adherent cell cultures. (**A**) Morphology of WG14 and L0125 cells in the presence of serum containing media (marked as FBS) or in the growth factors containing media (marked as sph). L0125 serves as a control cell line with stemness properties. (**B**) Representative immunoblots showing levels of GFAP, β-TUB III (differentiation markers) and OLIG2, SOX2, NANOG, OCT4 (stemness markers) in glioma primary cell cultures, NHA, NTERA, L0125 and L0627 cells. (**C**) Quantification of immunoblots. The level of a protein of interest in NHA equals 1 and is marked by a solid black line. β-ACTIN was used as a loading control. Statistical analysis was performed using a *t*-test comparing values in sph and FBS groups (* *p* < 0.05, *** *p* < 0.001), *n* ≥ 2, ±SD. (**D**) Expression of chosen differentiation (*GFAP, TUBBIII*) and stemness (*OLIG2, SOX2, NESTIN*) related genes in WG14 human patient-derived primary cells, L0125 and L0627 cells, cultured with FBS or with defined media (sph). The RT-qPCR data are shown as delta Ct values relative to the 18S expression. Statistical analysis was performed using a *t*-test (* *p* < 0.05, ** *p* < 0.01, *** *p* < 0.001), *n* ≥ 2, mean ± SD. The whole blots and molecular weight markers are shown in Supplementary Figure S10.

To obtain a subpopulation enriched in GSCs, cells were cultured at low density, without serum and in the presence of epidermal growth factor (EGF) and fibroblast growth factor (bFGF). We found that only two primary cell cultures, WG4 and WG14, were capable of forming spheres, as evidenced by using light microscopy (Figure 3A, Supplementary Figure S3) [51]. We have previously shown high expression of *NANOG*, *POU5F1*, *SOX2* and *PROM1* in WG4 spheres [51]. Here, we demonstrate that GCS-enriched spheres from WG14 expressed significantly higher levels of OLIG2 and lower levels of astrocytic (GFAP) and neuronal (β-Tubulin III) markers as compared with the adherent tumor cells (Figure 3B–D). Similar profiles were demonstrated in other GSCs originating from human GBMs (L0125 and L0627 cell lines) [49,59]. L0125 and L0627 spheres quickly attached to the cell cultures plates and branched out in serum-containing medium. Increased expression of GFAP and β-Tubulin III were observed upon the addition of serum containing media (Figure 3B–D). Interestingly, all studied glioblastoma-derived spheres (WG14, L0125, L0627) did not express NANOG or OCT4A, essential transcription factors that regulate self-renewal and pluripotency of embryonic stem cells [60]. NTERA-2 cells, a pluripotent human embryonic carcinoma cell line [61], were NANOG-, OCT4A- and SOX2-positive (Figure 3B,C). These results confirmed that GBM-derived spheres are lineage-restricted cells that could express some differentiation markers. Indeed, the presence of serum increased the levels of astrocytic and neuronal markers, and *NESTIN* and *SOX2* were highly expressed (Figure 3B–D).

### 3.3. EMT Markers/Regulators and Migratory Properties of Patient-Derived Glioma Cell Cultures

During epithelial-to-mesenchymal transition (EMT) cells acquire mesenchymal features, resulting in increased motility and invasiveness that are crucial for tumor progression [62]. Although gliomas do not undergo the classical EMT program, the majority of molecules involved in EMT play a role in the glial-to-mesenchymal transition, GMT [63], a process in which a subpopulation of glioma cells becomes highly motile and more resistant to treatment, and invades the brain parenchyma, generating micrometastases [64]. The analysis of RNA-seq data from TCGA databases showed significant upregulation of genes encoding mesenchymal markers and EMT regulators such as *CDH2* (coding N-cadherin), *VIM* (coding Vimentin), *SNAIs* and *TWISTs* in tumor samples, particularly in high-grade human gliomas (grade 4) and MES-GBMs (Supplementary Figure S4A,B). The *CDH1* expression (coding E-cadherin) was relatively low, and the lowest level was found in MES-GBMs (Supplementary Figure S4A,B). Patients with low expression of *CDH2*, *VIM*, *SNAIs* and *TWISTs* had longer survival compared with those of high expression (Supplementary Figure S4C). 

**Figure 4:**
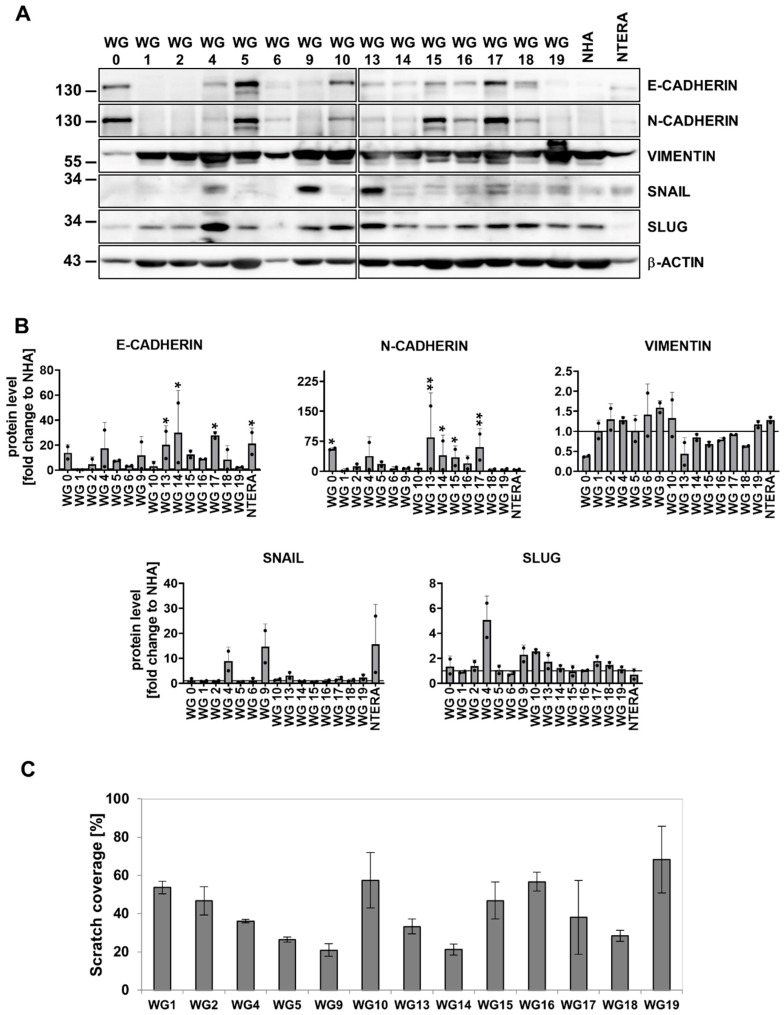
Epithelial mesenchymal transition (EMT) related genes and proteins in human primary glioma cell cultures. (**A**) Representative immunoblots showing the EMT markers levels in glioma primary cultures, NHA and NTERA cells. (**B**) Quantification of immunoblots. The level of a protein of interest in NHA cells equals 1 and is marked by a solid black line. β-ACTIN was used as a loading control. Statistical analysis was performed using one way ANOVA with Dunnett’s post hoc test comparing values obtained in tumor cells to NHA cells (* *p* < 0.05, ** *p* < 0.01,), *n* = 2, mean ± SD. (**C**) Quantification of glioma cell migration using an in vitro scratch assay. Results are presented as the percentage of scratch coverage after 18 h, *n* = 3, mean ± SD. The whole blots and molecular weight markers are shown in Supplementary Figure S11.

We analyzed the expression of EMT-related genes (top 30 genes from the web-based EMTome portal [65]) in GBM-derived cell cultures. The heatmap shows different expressions of the EMT gene signature in primary cell cultures (Supplementary Figure S5A). Interestingly, the mesenchymal markers VIMENTIN and CD44 were upregulated at mRNA and protein levels in all tested cell cultures (Figure 1F and Figure 4A, Supplementary Figure S5B). An elevated level of *CDH2* was found in GBM-derived cells (Supplementary Figure S5B). Immunoblot analysis showed higher levels of N-CADHERIN in the WG0, WG4, WG5, WG13, WG14, WG15 and WG17 cultures (Figure 4A,B). The expression of *CDH1* (an epithelial marker) was low in tested cell lines (Supplementary Figure S5B). The analysis of EMT-inducing transcription factors showed elevated levels of SNAIL in WG4, WG9, and WG13 cells, and similar levels of SLUG in most of the studied cell cultures, with the exception of WG4 cells having the highest SLUG protein level (Figure 4A,B). We determined the migratory properties of human GBM-derived cells using a scratch assay. A majority of cells (WG1, WG2, WG4, WG10, WG13, WG15, WG16, WG17 and WG19) closed 30–50% of the wound in less than 18 h (Figure 4C, Supplementary Figure S5C). These data show that the majority of GBM-derived cells have high migratory capacity.

### 3.4. Evaluation of Anti-Tumor Effects of TMZ or DOX on Human Glioma-Derived Cells

**Figure 5:**
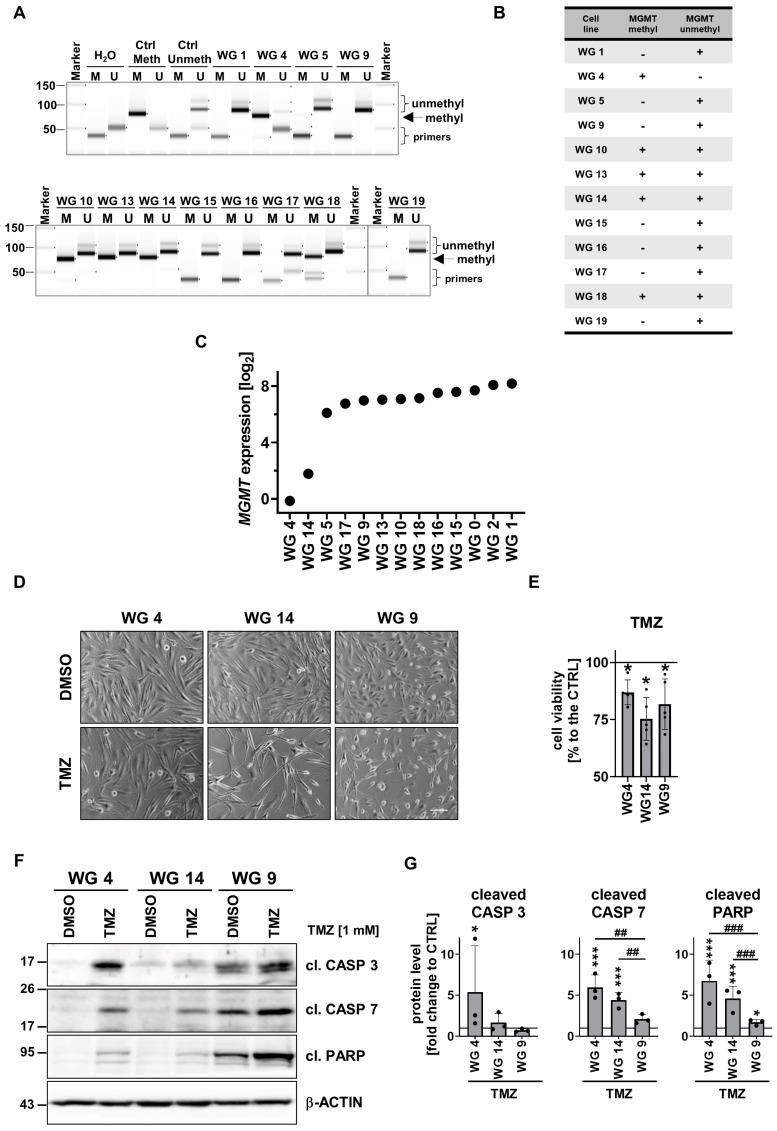
*MGMT* promoter methylation and the impact of TMZ on primary glioma cell cultures. (**A**) Evaluation of the *MGMT* promoter methylation in primary glioma cell cultures and (**B**) a summary table. (**C**) MGMT expression in primary glioma cell cultures based on RNA-seq analysis. (**D**) Microscopic images of WG4, WG14 and WG9 treated with TMZ (1 mM, 72 h). Scale bar: 100 µm. (**E**) Viability of WG4, WG14 and WG9 cells after TMZ treatment, determined by MTT metabolism test. The viability of untreated cells was set as 100% and marked with a black solid line. Statistical analysis was performed on raw data using a *t*-test in the comparison of treated to control groups (* *p* < 0.05,), *n* = 5, mean ± SD. (**F**) Representative immunoblots showing the levels of apoptotic proteins: cleaved caspase 3, cleaved caspase 7 and cleaved PARP (cl. CASP 3, cl. CASP 7, cl. PARP, respectively) in TMZ-treated WG4, WG14 and WG9 cells. (**G**) Quantification of immunoblots. The level of a protein of interest in control cells equals 1 and is marked by a solid black line. β-actin was used as a loading control. Statistical significance was determined by one-way ANOVA followed by Dunnett’s post hoc test in the comparison of treated to untreated cells (* *p* < 0.05, *** *p* < 0.001) or between the cell lines (## *p* < 0.01, ### *p* < 0.001), *n* = 3, mean ± SD. The whole blots and molecular weight markers are shown in Supplementary Figure S12.

We analyzed the *MGMT* promoter methylation in 12 cell cultures by using methylation-specific (MS)-PCR. The unmethylated *MGMT* gene promoter was found in seven cell cultures (WG1, WG5, WG9, WG15, WG16, WG17 and WG19 cells), whereas the methylated *MGMT* promoter was detected only in WG4 cells. The intermediate status of the *MGMT*, both methylated and unmethylated, was found in WG10, WG13, WG14 and WG19 cell cultures (Figure 5A,B). These results were corroborated by *MGMT* expression data (Figure 5C). The lowest expression was found in WG4 cells, intermediate in WG14 cells and high in WG9 cells and other tested cells. 

We tested the effects of 1 mM TMZ on GBM-derived cells with different statuses of the *MGMT* gene promoter: WG4 (*MGMT* low), WG14 (*MGMT* intermediate) and WG9 (*MGMT* high). The viability of glioma cells after 72 h treated with TMZ was significantly reduced to 75% (Figure 5D,E). The strongest accumulation of apoptosis markers—cleaved caspase 3 and caspase 7 and fragments of PARP—was found in TMZ-treated WG4 cells. The cytotoxic effect of TMZ was less prominent in WG14 cells, whereas WG9 cells were resistant to the treatment (Figure 5F,G).

Next we tested how the cells responded to doxorubicin (DOX) added at different concentrations (50–1000 nM) for 48 h (Supplementary Figure S7). DOX reduced cell viability in a dose-dependent manner, with 25% of WG9 cells and 50% of WG4 and WG14 cells killed after drug administration. These results showed that WG9 primary cultures were more resistant to DOX. The half-maximal-effective concentration (EC_50_) values were calculated for WG4, WG14 and WG9 cells and were 1.09, 1.47 and 1.85 mM, respectively (Supplementary Figure S7). Evaluation of caspase-cascade protein levels showed that WG4 and WG14 cells were more sensitive to the drug than WG9 cells (Figure 7A,B). Altogether, the results support the notion that *MGMT* promoter methylation defines cell responses not only to TMZ but also DOX treatment. 

### 3.5. Activation of EGFR Signaling Pathway Modifies the Response of Human Glioma-Derived Cells to TMZ and DOX 

**Figure 6:**
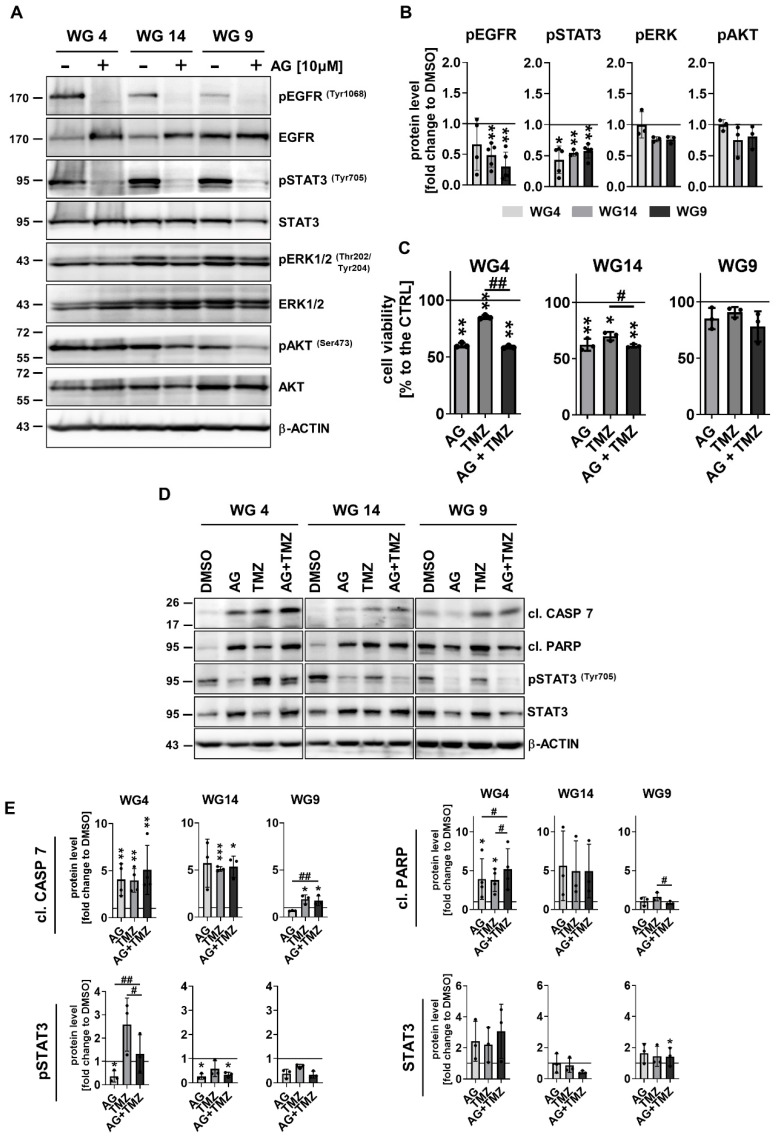
Treatment with the EGFR inhibitor AG1478 modifies sensitivity of glioma cells to TMZ. (**A**) Representative immunoblots of phosphorylated proteins involved in EGFR signaling pathways in WG4, WG14 and WG9 cells in the presence (6 h) and absence of 10 µM AG1478 (AG). (**B**) The densitometric quantification. The level of a protein of interest in control cells equals 1 and is marked by a solid black line. β-ACTIN was used as a loading control. Statistical significance was determined by a t-test in comparison of treated to non-treated cells (* *p* < 0.05, ** *p* < 0.01, *n* ≥ 3, mean ± SD. (**C**) Viability of WG4, WG14 and WG9 cells after 10 µM AG alone or combined with 1 mM TMZ (AG + TMZ) for 72 h was determined with a PrestoBlue test. Viability of the control group was set as 100% and marked by a black solid line. Statistical significance was determined on raw data by one-way ANOVA followed by Dunnett’s post hoc test in the comparison of treated to untreated cells (* *p* < 0.05, ** *p* < 0.01), or by one-way ANOVA followed by uncorrected Fisher’s LSD test between the groups: AG or TMZ vs. AG + TMZ (# *p* < 0.05, ## *p* < 0.01), *n* = 3, mean ± SD. (**D**) Representative immunoblots detecting the apoptosis markers: cleaved caspase 7 and cleaved PARP (cl. CASP 7, cl. PARP, respectively), and phospho-STAT3 (pSTAT3) and STAT3 of WG4, WG14 and WG9 cells treated with 10 µM AG, 1 mM TMZ or with a combination of AG + TMZ for 72 h. (**E**) Quantification of immunoblots. The level of protein of interest in control cells equals 1 and is marked by a solid black line. β-ACTIN was as a loading control. Statistical significance was determined by one-way ANOVA followed by Dunnett’s post hoc test in comparison of treated to untreated control cells (* *p* < 0.05, ** *p* < 0.01, *** *p* < 0.001) or by one-way ANOVA followed by uncorrected Fisher’s LSD test between the groups: AG or TMZ vs. AG + TMZ (# *p* < 0.05, ## *p* < 0.01), *n* ≥ 3, mean ± SD. The whole blots and molecular weight markers are shown in Supplementary Figures S13 and S14.

Due to the minor effect of TMZ or DOX alone on glioma cell viability, we explored whether blocking EGFR signaling with the specific inhibitor AG1478 (AG) would modify the cell response to the drugs. First, the effects of inhibition of EGFR on glioma cells were analyzed (Figure 6A,B).

AG efficiently blocked EGFR activation in WG4, WG14 and WG9 cells was evidenced by a reduction of phospho-EGFR levels (Figure 6A,B). Interestingly, treatment with AG for 6 h resulted in the reduction of phospho-STAT3 levels without significant decrease phospho-ERK and phospho-AKT levels, and altering total ERK and AKT levels (Figure 6A,B; Supplementary Figure S6B). Cell viability was not affected after exposure to AG for 6 h (Supplementary Figure S6A); however, longer exposure (72 h) significantly reduced the viability of WG4 and WG14 cells (Figure 6C). To study whether AG sensitizes to TMZ-induced cell death, we calculated the effect size (Hedge’s g) for single AG or TMZ treatment, and combined AG + TMZ treatment. Hedge’s g = 0.2, 0.5 and 0.8 are often cited as indicative of a small, medium and large effect, respectively. The effects for WG4 were as follows: AG (4.5), TMZ (1.5), AG and TMZ (4.9); on WG14: AG (3.0), TMZ (2.7), AG and TMZ (3.6); and on WG9: AG (0.3), TMZ (0.2), AG + TMZ (0.5). Notably, in the WG4 and WG14 cells we observed additive effects of AG + TMZ and decreased cell viability, whereas WG9 cells were the most resistant to the treatments. The analysis of apoptosis markers confirmed that the combined treatment with AG + TMZ resulted in the strongest accumulation of cleaved caspase 7 and PARP in WG4 and WG14 cells in comparison with WG9 cells. The additive effect of AG + TMZ was observed in WG4 cells (Figure 6D,E). While phospho-STAT3 levels were reduced in AG + TMZ-treated cells, levels of phospho-ERK and phospho-AKT were not affected in treated cells (Supplementary Figures S6D,E and S17 for whole Western Blots).

**Figure 7:**
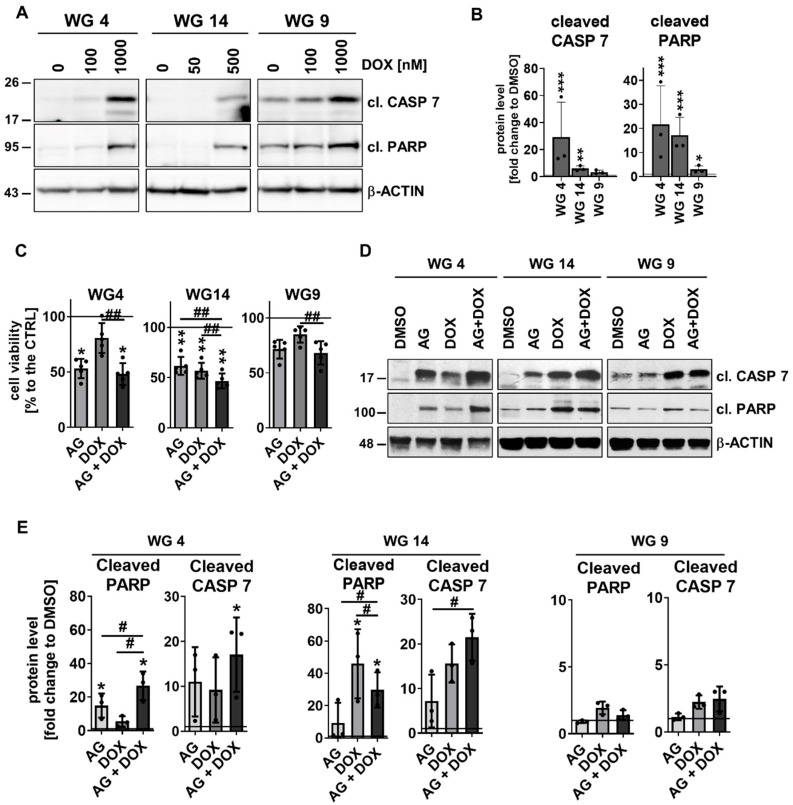
AG1478-enhanced cytotoxicity exerted by DOX in primary glioma cell cultures. (**A**) Representative immunoblots showing the apoptosis markers: cleaved caspase 7 and cleaved PARP (cl. CASP 7, cl. PARP, respectively) in WG4, WG14 and WG9 cells treated with DOX for 48 h. (**B**) Densitometric quantification of effects of DOX at the high doses: 0.5 and 1 mM. The level of a protein of interest in control cells equals 1 and is marked by a solid black line. β-ACTIN was used as a loading control. Statistical significance was determined by one-way ANOVA followed by Dunnett’s post hoc test (* *p* < 0.05, ** *p* < 0.01, *** *p* < 0.001), *n* = 3, mean ± SD. (**C**) Cell viability of WG4, WG14 and WG9 cells after 10 µM AG, 0.5 mM DOX or combined AG + DOX treatment for 48 h, determined by PrestoBlue test. Viability of the control group was set as 100% and marked by a black solid line. Statistical significance was determined on raw data by one-way ANOVA followed by Dunnett’s post hoc test in comparison to untreated control cells (* *p* < 0.05, ** *p* < 0.01, *** *p* < 0.001) or by one-way ANOVA followed by uncorrected Fisher’s LSD test between the groups: AG or DOX vs. AG + DOX (## *p* < 0.01), *n* ≥ 3, mean ± SD. (**D**) Representative immunoblots detecting the apoptosis markers: cleaved caspase 7 and cleaved PARP (cl. CASP 7, cl. PARP, respectively) of WG4, WG14 and WG9 cells treated with 10 µM AG, 0.5 mM DOX or with a combination of AG + DOX for 48 h with (**E**) the densitometric quantification. The level of a protein of interest in control cells equals 1 and is marked by a solid black line. Statistical significance was determined by one-way ANOVA followed by Dunnett’s post hoc test in comparison to untreated control cells (* *p* < 0.05) or by one-way ANOVA followed by uncorrected Fisher’s LSD test between the groups: AG or DOX vs. AG + DOX (# *p* < 0.05), *n* = 3, mean ± SD. The whole blots and molecular weight markers are shown in Supplementary Figures S15 and S16.

Similar changes were detected after the AG + DOX treatment (Figure 7C). We calculated the effect size (Hedge’s g) for all of the treatments to determine the effectiveness of the treatments. The effects on WG4 were as follows: AG (1.7), DOX (0.7), AG + DOX (1.8); on WG14: AG (2.0), DOX (2.3), AG + DOX (2.9); and on WG9: AG (0.8), DOX (0.4), AG + DOX (1.0). An additive effect between AG and DOX was visible in the tested cell lines, with a minor effect on WG9 cells. Markedly, the analysis of cell death markers by Western blotting confirmed those results (Figure 7D,E). The accumulation of cleaved caspase 7 and cleaved PARP was found in WG4 and WG14 cells but not in WG9 cells.

## 4. Discussion

We introduced small modifications into existing protocols [48,66] that resulted in producing a simple procedure to culture both adherent cells and GSCs derived from glioma patient tumor samples. We generated 13 adherent cell cultures out of 16 GBM specimens, including two sphere cultures, enriched in GSCs. We established two cell lines from a WHO grade 1 tumor, and two cell lines from a WHO grade 2/3 tumor, which is a rare event, as lower-grade patient-derived glioma (LGG) is challenging [67]. Virtually all LGG cell lines generated to date from adult patients represent oligodendroglioma WHO grade 3 [68,69,70]. The developed GBM-derived cell cultures were more similar to high- grade gliomas from TCGA than to LGG in terms of transcriptional profiles. 

The low frequency of establishing GSC-enriched spheres likely stems from the fact that only the WG4 and WG14 cells had a high expression of neural stem and precursors markers responsible for self-renewal of GSCs (*OLIG2*, *SOX2* and *NESTIN)*, while embryonic stem cell markers (NANOG and OCT4A) were not expressed [60]. This low frequency is in agreement with recent findings showing that a significant part of GBM samples did not form long-term serum-free cell cultures [19,71]. The expression of astrocytic (*GFAP*, *S100*) and neuronal (*MAP2*, *TUBB3*) markers in the same cells suggests that GBM-derived spheres undergo aberrant differentiation, as was shown previously [72].

The detected gene expression profiles and expressed proteins reveal a high degree of intertumoral heterogeneity among GBM-derived cell cultures, representing mixed subtypes within each GBM-derived cell culture. Indeed, Patel et al., using single cell RNA sequencing, reported that distinct cells in the same tumor exhibit transcriptional programs from distinct subtypes [18]. GBM cells exist in four main cellular states and show state transition, or plasticity [73]. WG4 and WG14 cells expressed PN and CL signatures and, consistently with CL and PN subtypes, were highly proliferating cells.

Transcriptional analysis is not routinely feasible in clinical setting, therefore a simplified method based on the expression of some proteins was proposed. We evaluated seven proteins specific to distinct GBM subtypes: PN (OLIG2, IDH1^R132H^, TP53 and PDGFRα), CL (EGFR) and MES (CHI3L1/YKL40, CD44 and phospho-STAT3). *EGFR* alterations were found in 55% of patients in the TCGA-GBM dataset. It was also found in most of the presented cultures, with high levels of phosphorylated EGFR in WG4, WG9 and WG14 cells. WG4, WG14 and WG17 cells were positive for the mutant IDH1^R132H^. Increased TP53 protein levels in WG4, WG14 and WG17 cells suggests non-functional TP53, as wild-type TP53 is rapidly degraded and mutant forms are stabilized in tumor cells [74]. Mutation in the *TP53* gene was confirmed by deep sequencing in WG4 cells. This observation is consistent with recent reports [16,23] showing that WHO grade 4 gliomas with *IDH1* mutations harbor *TP53* mutations. WG13 and WG17 cells were assigned with the MES subtype and analysis of EMT proteins showed that a majority of cells express elevated level of VIMENTIN, CD44 and SLUG. High expression of CHI3L1/YKL40 was found only in WG4 and WG14 cells. Recent findings indicate that CHI3L1/YKL40 is highly expressed only in a small fraction of the primary tumor samples [75]. N-CADHERIN, SNAIL and phospho-STAT3 levels varied in cell cultures. The acquisition of mesenchymal phenotypes by a majority of cultures could be due to clonal selection under in vitro conditions [71,76]. Our results demonstrate that using a small set of markers, we can define major GBM subtypes and the patient-derived cell cultures recapitulate the GBM heterogeneity of GBM, although gene expression was not fully recapitulated at protein levels. Indeed, Brennan et al. using a targeted proteomic profiling showed that the impact of specific genomic alterations on downstream pathway signaling is not linear and not always concordant with a genotype [23].

The classification of GBM may assist in selecting therapies, for example CL-GBMs are more responsive to radiation and chemotherapy, as typically, intact TP53 may control DNA-damage-induced cell death. The MES subtype is the most aggressive and strongly associated with a poor prognosis when compared to the PN subtype [77], and a shift from a PN to an MES subtype can occur in patients following radiotherapy and chemotherapy [78].

EGFR-targeted therapy has attracted much attention due to frequent alterations in malignant gliomas. A specific EGFR inhibitor AG1478 [N-(3-chlorophenyl)-6,7-dimethoxyquinazolin-4-amine] competitively binds to the ATP pocket of EGFR and inhibits its activity [79,80]. Previous data showed anti-proliferative effects of AG1478 and enhancement of sensitivity to cytotoxic drugs, such as cisplatin, etoposide and DOX in different cancer cells [81,82], including human EGFRvIII glioma cells in vivo [83,84] that resulted in preclinical studies [85].

We used molecularly diverse GBM-derived cell cultures with *MGMT* promoter methylation to determine if inhibition of EGFR with AG1478 would sensitize GBM cells to TMZ or DOX. We found that AG-treated WG4 cells (with methylated *MGMT* promoter) or WG14 cells (with intermediate *MGMT* methylation status) were more sensitive to DOX than WG9 cells (with the unmethylated *MGMT*). AG1478 + TMZ resulted in the strongest accumulation of apoptosis markers in WG4 cells. Our data suggest that *MGMT* promoter methylation could predict the response not only to TMZ but also to DOX. While TMZ and DOX induce different DNA damage responses, and most alkylating drugs (TMZ) are sensitive to MGMT-mediated repair, other studies showed that *MGMT* promoter methylation affects responses to radiotherapy and other cytotoxic drugs [4,86,87]. A recent systematic review and meta-analysis confirmed a *MGMT* methylation status as a clinical biomarker in GBM patients, showing association with better overall and progression-free survival in patients treated with alkylating agents [88] and tyrosine kinase inhibitors [89]. The mechanism of action of DOX is known and has been described as independent from *MGMT* promoter status; however, recent studies link the MGMT expression with the response to non-alkylating drug treatment [90,91].

AG1478-induced blockade of EGFR signaling shows that the levels of active, phosphorylated STAT3 were reduced without changing phospho-AKT and ERK levels. Phosphorylation of STAT3 requires nuclear entry of EGFRvIII and formation of an EGFRvIII-STAT3 nuclear complex [92]. An inactive PI3K-AKT pathway could result from PTEN alterations, mutations within the gene encoding the p110 catalytic subunit of PI3K (*PIK3CA*), *AKT* amplification [93] or pathway activation via PDGFRs [94]. Indeed, DNA sequencing analysis revealed mutations in *PTEN* and *PIK3CA* genes in WG14 and WG9 cells, respectively (data not shown). Our results show that EGFR inhibition via reducing STAT3 phosphorylation sensitize some GBM cells to the treatment with TMZ or DOX, providing a new modality for those chemotherapeutics resistant cells.

DOX is a cytotoxic, anti-cancer drug with well-known pharmacokinetics that, due to low penetration of the BBB and serious side effects, is not used in GBM therapy. However, many new formulations of DOX with nanoparticles and liposomes may improve its delivery to glioblastomas [14,15,16,17,18]. Interestingly, co-delivery of DOX and EGFR siRNA in intracranial U87MG xenografts prolonged the life span of glioma-bearing mice and induced apoptosis in gliomas [11], which is exactly the combination we recommend based on our results. Disrupting the peritumoral BBB with focused ultrasound (FUS) and interstitial thermal therapy (LITT) improves the accumulation of DOX in GBM-bearing mice [12]. LITT combined with low-dose DOX results in longer survival of recurrent GBM patients. Low doses of DOX were safe for patients, even with extended (>6 weeks) dosing [95], and showed promising results in a phase I trial (GBM-LIPO trial) in which patients with relapsed glioblastoma harboring an *EGFR* amplification were treated with anti-EGFR doxorubicin-loaded immunoliposomes (anti-EGFR ILs-DOX) [46]. These results support using a combinatorial approach in well-defined GBM-derived cell cultures and advocate for the use of DOX together with EGFR-targeted therapy for GBM-patients. The results show that such a combination would be effective in GBM patients with amplified/mutated EGFR and with the methylated *MGMT* promoter.

## 5. Conclusions

The present study provides a simplified protocol to generate glioma-patient-derived cultures and glioma stem-like spheres, expressing to some extent features and subtypes of the original tumors. We provided transcriptional and marker-protein-based characterization of the cell cultures that allowed classification into major subtypes. We determined the response of WG4, WG9 and WG14 cells bearing the different *MGMT* promoter methylation to various cytotoxic drugs and evaluated a potential additive effect of EGFR blockade. Our results show that EGFR inhibition via reducing STAT3 phosphorylation sensitizes some GBM cells to treatment with TMZ or DOX, providing a new modality for those chemotherapeutic-resistant cells. We emphasized the need for proper characterization of the cells to obtain the most reliable results, especially when the new drugs or their combinations will be tested for clinical settings.

## Data Availability

The data were deposited to the European Genome-phenome Archive EGA (http://www.ebi.ac.uk/ega/, accessed on 13 January 2023), under accession numbers EGAS00001006849, EGAD00001009795.

## Supplementary Materials

The following supporting information can be downloaded at: https://www.mdpi.com/article/10.3390/cancers15051562/s1, Figure S1: Information about patient cohort and corresponding primary cell cultures. Figure S2: Immunofluorescent staining of selected proteins in primary cell cultures. Figure S3: Establishment of primary cell cultures and passaging. Figure S4: Expression of EMT-related gene in TCGA gliomas and the survival analysis of GBM patients with different expression of EMT-related genes. Figure S5: Characterization of the mesenchymal phenotype of primary glioma cell cultures. Figure S6: The effect of AG1478 alone and in combination with TMZ. Figure S7: Impact of DOX treatment to primary glioma cell cultures. Figures S8–S17: Whole blots related to the main figures. Table S1: Antibodies and reagents used.
