# Supplementary material for: Exploring Novel Therapeutic Opportunities for Glioblastoma Using Patient-Derived Cell Cultures
Source: Cancers (Basel). 2023 Mar 2;15(5):1562. doi: 10.3390/cancers15051562 (PMC10000883; doi:10.3390/cancers15051562)
Supplement: Supplementary file 1 [file cancers-15-01562-s001.zip › cancers-2196789-supplementary.pdf]

	Cell line	Histology (WHO grade)	Age	Sex
1	**WG 0**	*Glioblastoma (WHO G4)*	74	Male
2	**WG 1**	*Glioblastoma (WHO G4)*	68	Male
3	**WG 2**	*Glioblastoma (WHO G4)*	-	Male
4	**WG 3**	*Glioblastoma (WHO G4)*	44	Female
5	**WG 4**	*Glioblastoma (WHO G4)*	49	Male
6	**WG 5**	*Glioblastoma (WHO G4)* – regrowth	25	Female
7	**WG 6**	*Glioblastoma (WHO G4)*	66	Female
8	**WG 7**	*Pilocytic astrocytoma (WHO G1)*	15	Female
9	**WG 8**	*Pilocytic astrocytoma (WHO G1)*	14	Female
10	**WG 9**	*Glioblastoma (WHO G4)*	56	Female
11	**WG 10**	*Glioblastoma (WHO G4)*	46	Female
12	**WG 11**	*Oligodendroglioma anaplasticum (WHO G3)*	41	Female
13	**WG 12**	*Astrocytoma (WHO G2)*	31	Female
14	**WG 13**	*Glioblastoma (WHO G4)*	62	Male
15	**WG 14**	*Glioblastoma (WHO G4)*	70	Male
16	**WG 15**	*Glioblastoma (WHO G4)*	79	Male
17	**WG 16**	*Glioblastoma (WHO G4)*	34	Female
18	**WG 17**	*Glioblastoma (WHO G4)*	74	Male
19	**WG 18**	*Glioblastoma (WHO G4)*	48	Male
20	**WG 19**	*Glioblastoma (WHO G4)*	59	Male

Figure S1. Information about patient cohort and corresponding primary cell cultures
Clinical data included histological type, age, gender of patients with corresponding primary glioma cell cultures.

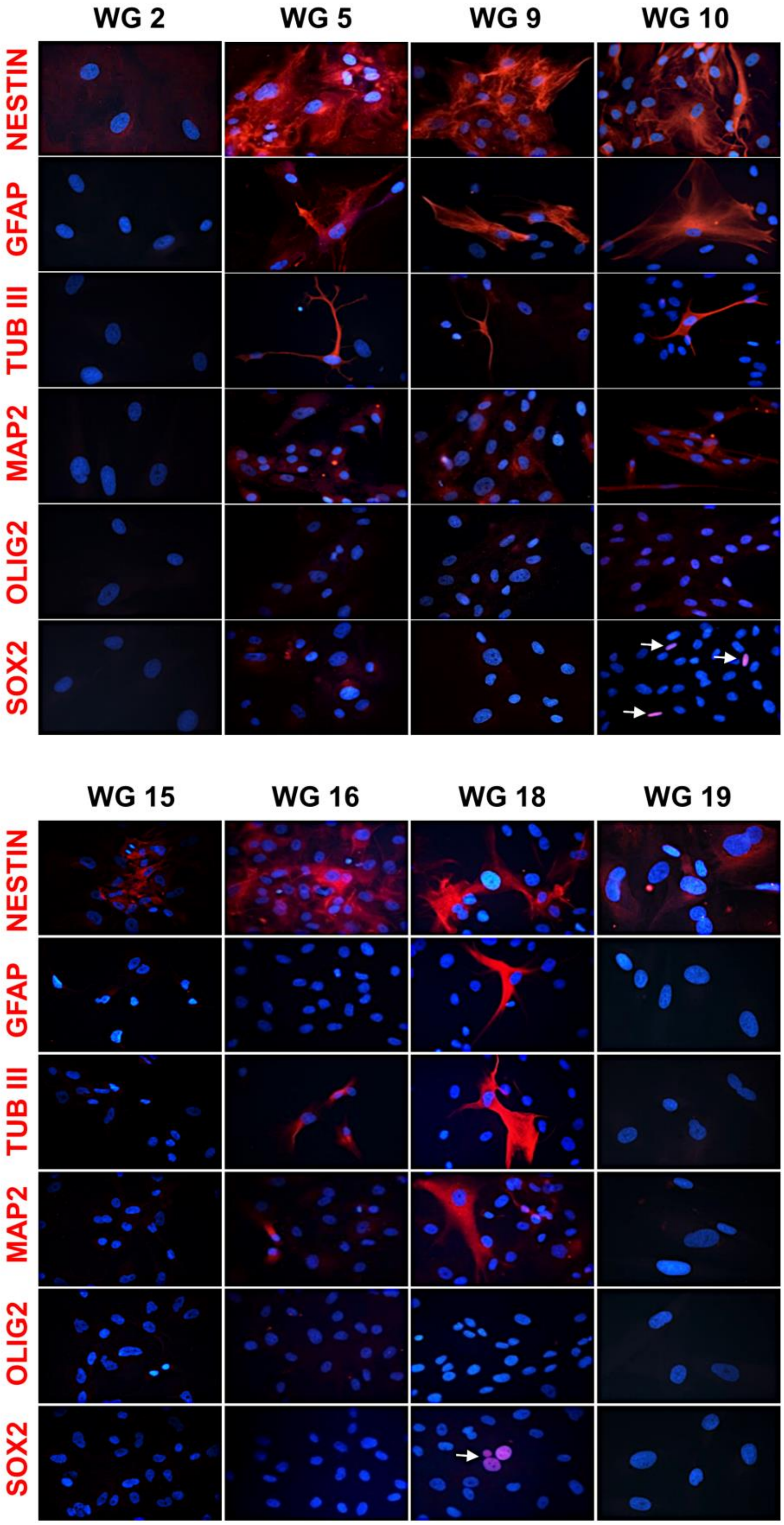

Figure S2. Immunofluorescent staining of selected proteins in primary cell cultures
Representative immunofluorescent staining of the stemness (NESTIN, SOX2, OLIG2) and differentiation (GFAP, β-TUB III, MAP2) markers in WG2, WG5, WG9, WG10, WG15, WG16, WG18 and WG19 cells. White arrows indicate SOX2 nuclear staining. Scale bar: 100 μm.

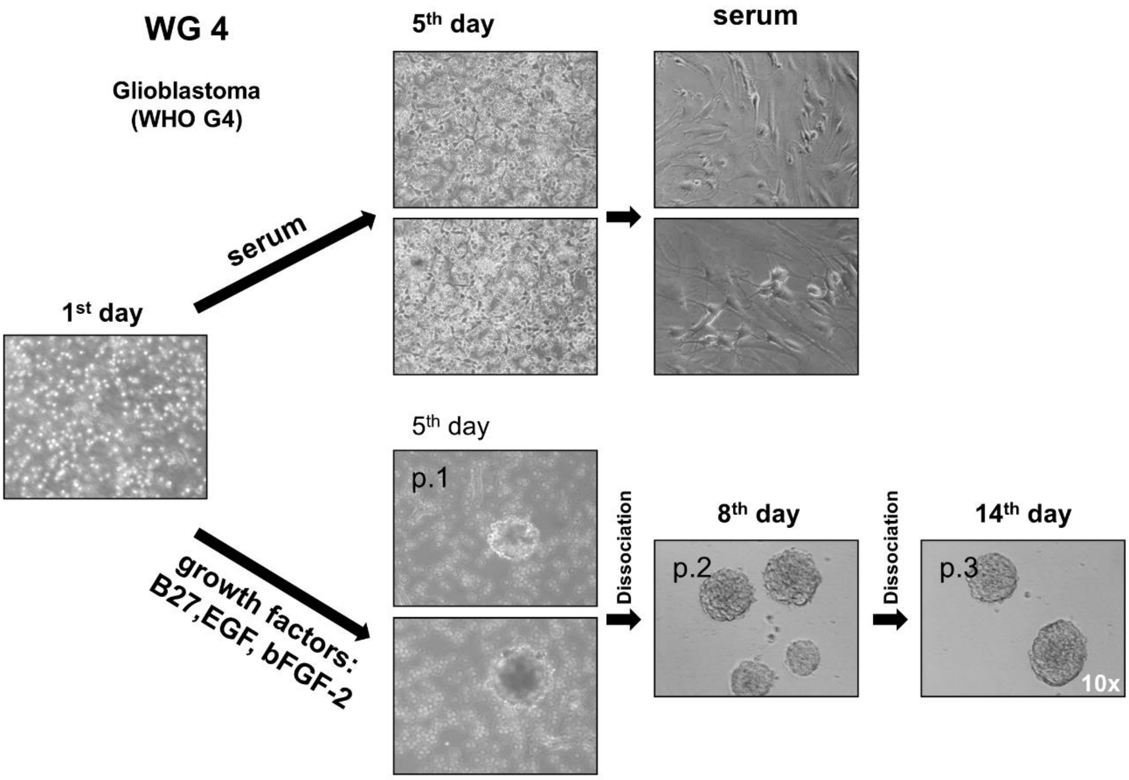

Figure S3. Establishment of primary cell culture and passaging

Light microscopy images of dissociated WG4 cells 1 day after tissue dissection, and after 5 days in the presence of serum (FBS) or under sphere conditions (growth factors). Morphology of adherent WG4 cells growing in the presence of serum, and second and tertiary generation of WG4 neurospheres growing in a medium supplemented with B27 and growth factors.

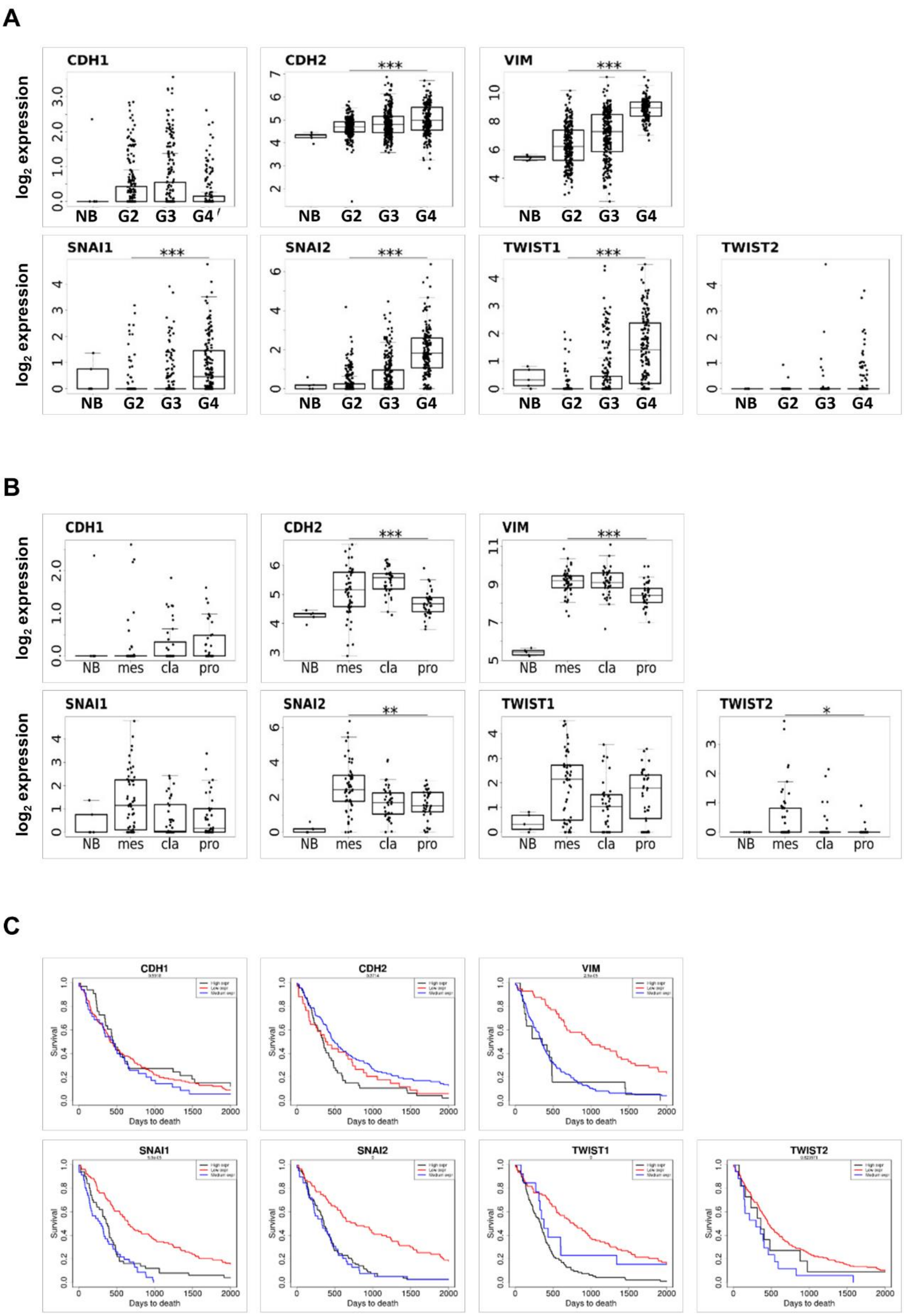

Figure S4. Expression of EMT-related genes in TCGA gliomas and the survival analysis of GBM-patients with different expression of EMT-related genes

(A) Expression of key EMT-related genes in glioma of different grades. Boxplots show the expression of *CDH1*, *CDH2*, *VIM*, *SNAIs*, *TWISTs* in normal brain samples (NB) compared to human gliomas of different grades (G2-G4) in the TCGA dataset. **(B)** Expression of key EMT genes in different subtypes of GBM. Boxplots show the expression of *CDH1*, *CDH2*, *VIM*, *SNAIs*, *TWISTs* in normal brain samples (NB) compared to different subtypes of GBM (mes-mesenchymal, cla-classical, pro-proneural). **(C)** Survival analysis of glioma patients with different expression of key EMT genes in the TCGA dataset. Association of *CDH1*, *CDH2*, *VIM*, *SNAIs* and *TWISTs* expression with overall survival in all grades gliomas in the TCGA dataset (red line - low expression, black line – high expression, blue line - medium expression).

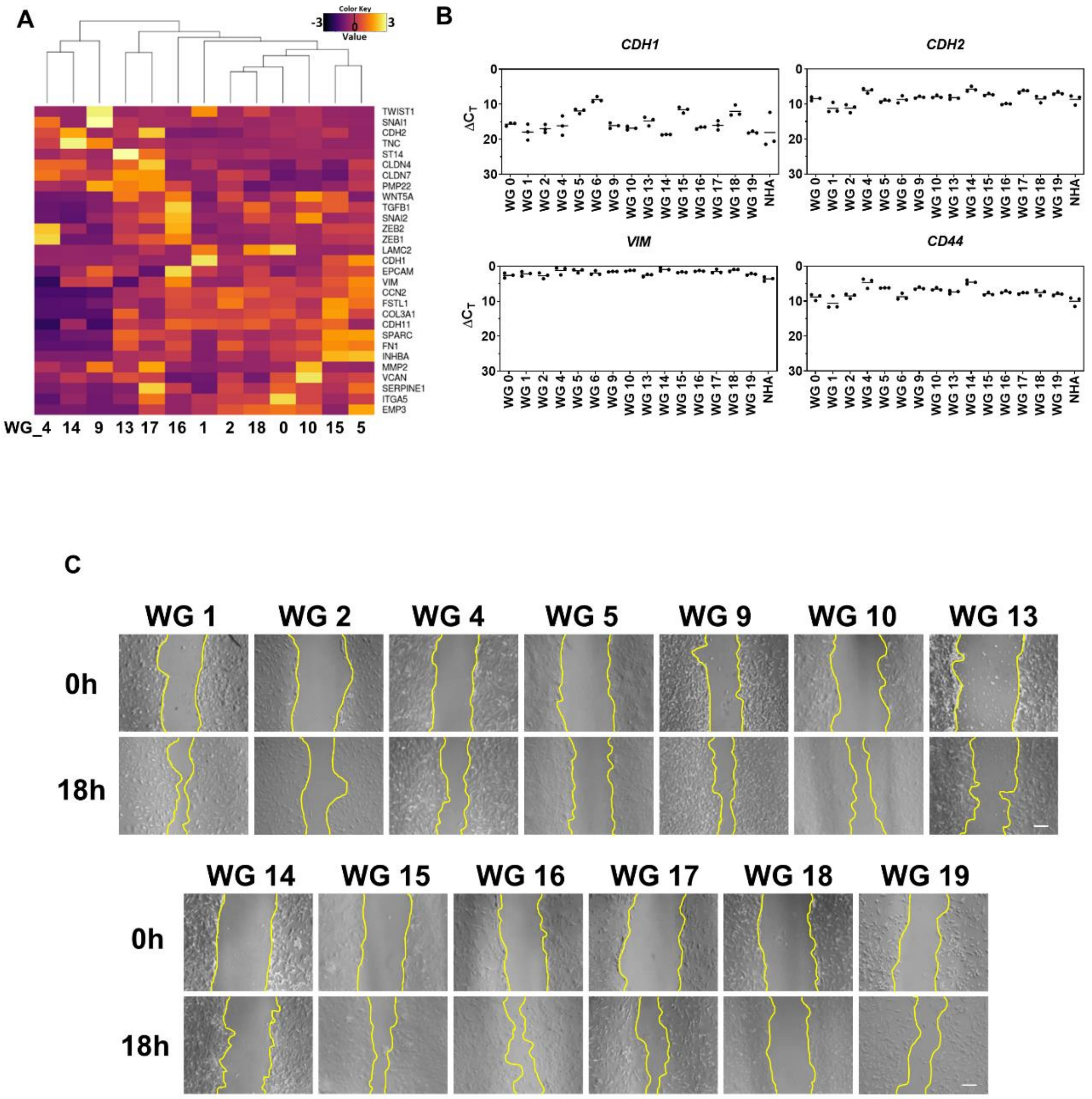

Figure S5. Characterization of the mesenchymal phenotype of primary glioma cell cultures

(A) RNAseq of primary cell lines represented as a heatmap of EMT genes across primary glioma cell cultures. **(B)** Expression of chosen EMT-related genes (*CDH1, CDH2, VIM, CD44*). The RT-qPCR data are shown as delta Ct values relative to the 18S expression. Statistical analysis was performed using t-test (*p<0.05, **p<0.01, ***p<0.001), n=3, mean ± SD. **(C)** The migratory ability of different primary glioma cells was analyzed by scratch assay. Photos represent wound closure after 0 h and 18 h. Scale bar: 200 µm.

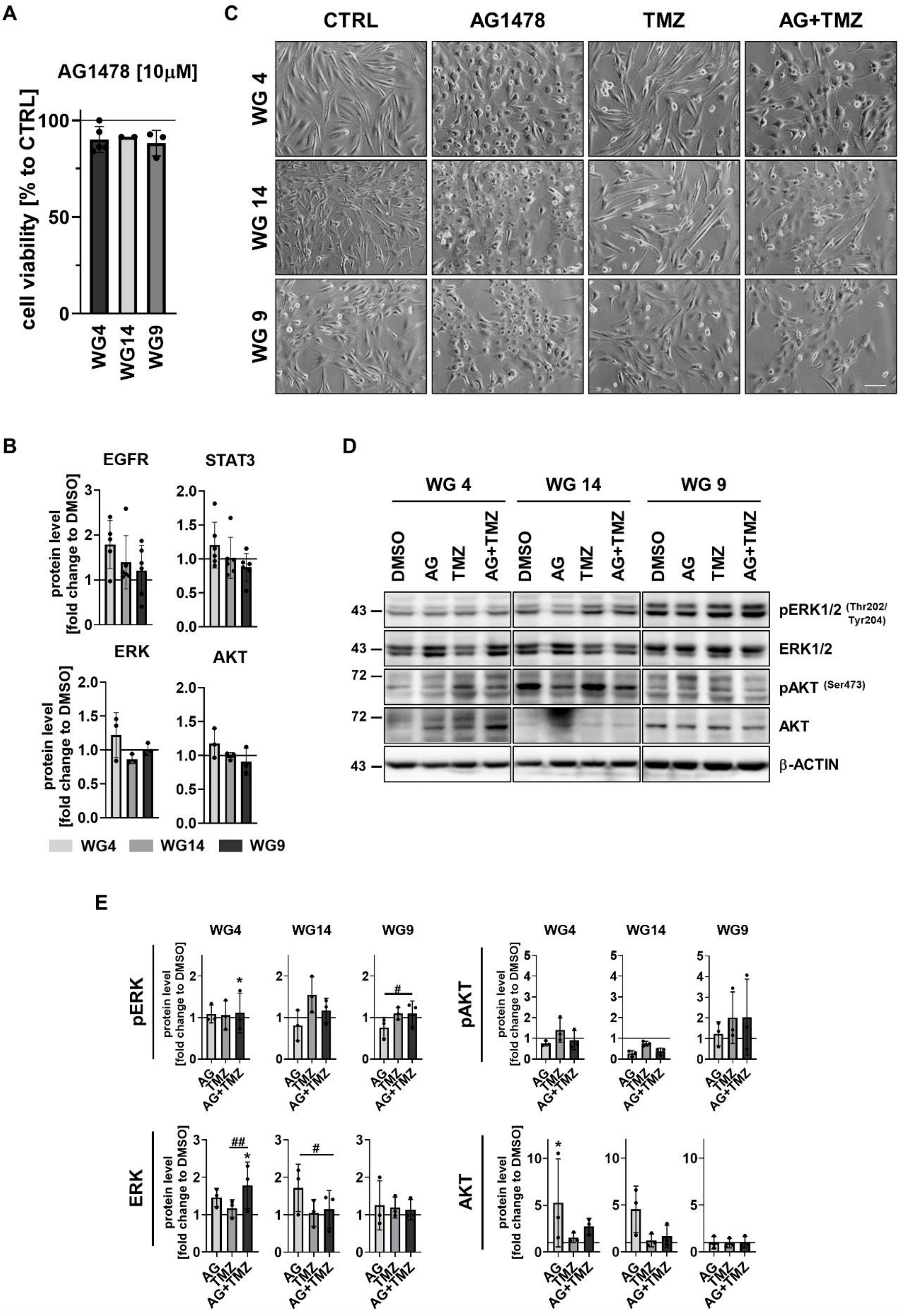

Figure S6. The effect of AG1478 alone or in combination with TMZ

(A) Cell viability of WG4, WG14 and WG9 cells after treatment with 10 μM AG for 6 h, determined by PrestoBlue test. Viability of the control group was set as 100% and marked by a black solid line. Statistical significance was calculated on raw data by t-test in comparison to untreated cells (not significant). **(B)** The densitometric quantification of total: EGFR, STAT3, ERK and AKT in cells exposed to AG (10 μM) for 6 h. The level of a protein of interest in control cells equals 1 and is marked by a solid black line. β-ACTIN was used as a loading control. Statistical significance was determined by t-test in comparison to control conditions (without AG), (not significant), n≥3, mean ± SD. **(C)** Representative images of WG4, WG14 and WG9 cells after 10 μM AG, 1 mM

TMZ and combined AG+TMZ treatment for 72 h. Scale bar: 100 μm. **(D)** Representative immunoblots of proteins involved in EGFR signaling pathways in WG4, WG14 and WG9 cells treated with 10 μM AG, 1 mM TMZ or with combination of AG+TMZ for 72 h with **(E)** the densitometric quantification. The level of a protein of interest in control cells equals 1 and is marked by a solid black line. β-ACTIN was used as a loading control. Statistical significance was determined by one-way ANOVA followed by Dunnett's post hoc test in comparison to untreated control cells (*p<0.05, **p<0.01, ***p<0.001) or by one-way ANOVA followed by uncorrected Fisher's LSD test between the groups: AG or TMZ vs AG+TMZ (#p<0.05, ##p<0.01, ###p<0.001), n=3, mean ± SD.

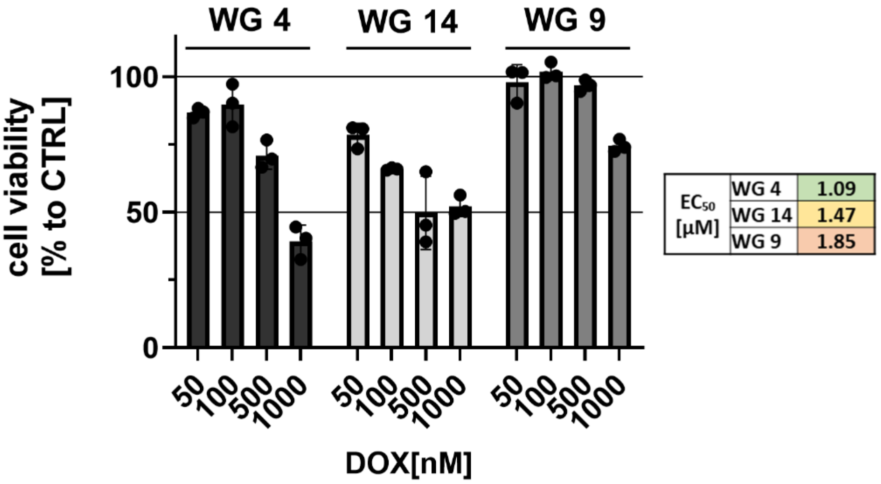

Figure S7. Impact of DOX treatment to primary glioma cell cultures
(A) Cell viability of WG4, WG14 and WG9 cells after DOX treatment for 48 h, determined by MTT cell metabolism test. The viability of untreated control cells was set as 100% and marked with a black solid line. EC_{50} was calculated using a linear relationship between the dose and cell viability. n=3, mean ± SD.

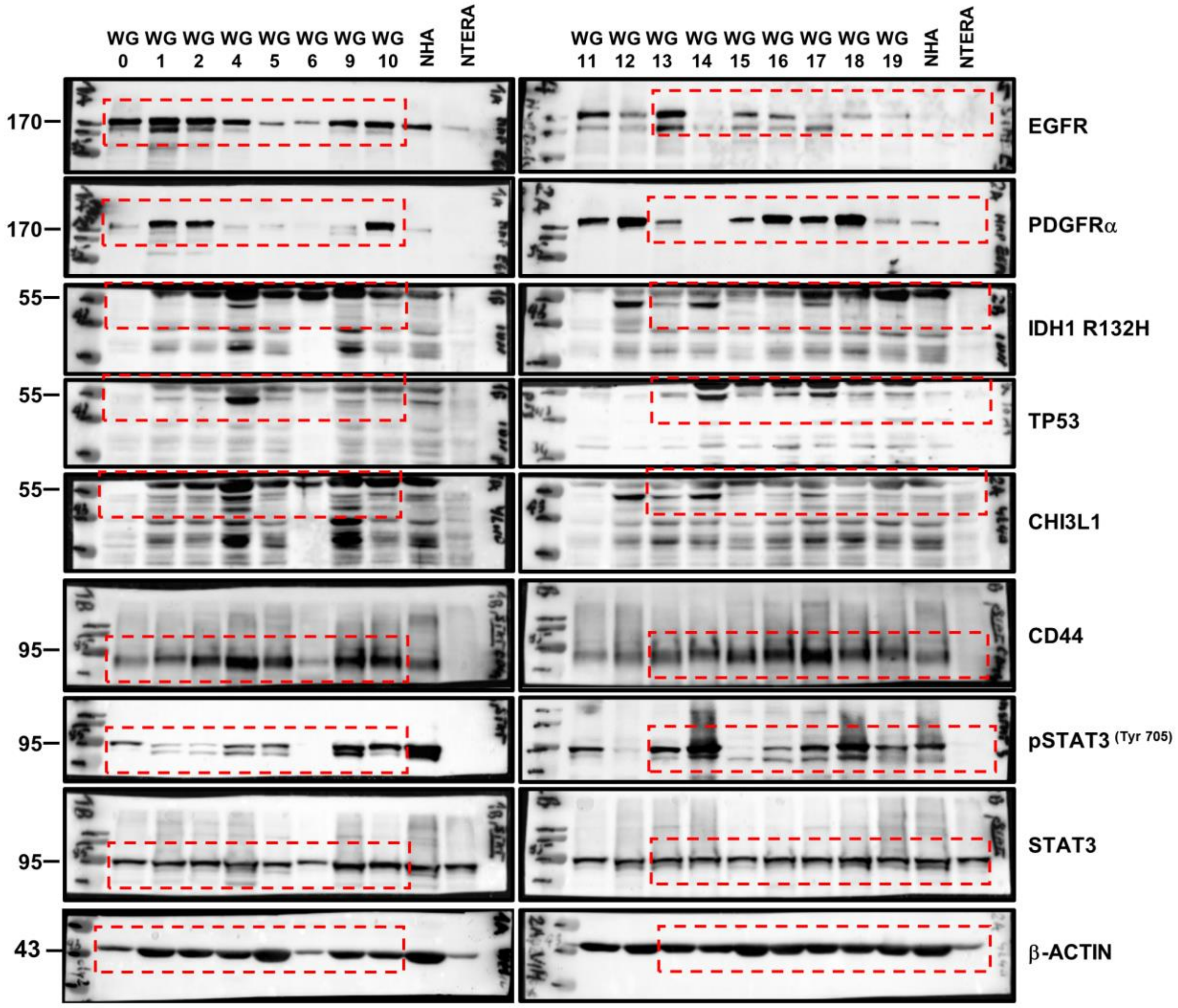

Figure S8. Western Blots related to Figure 1F.

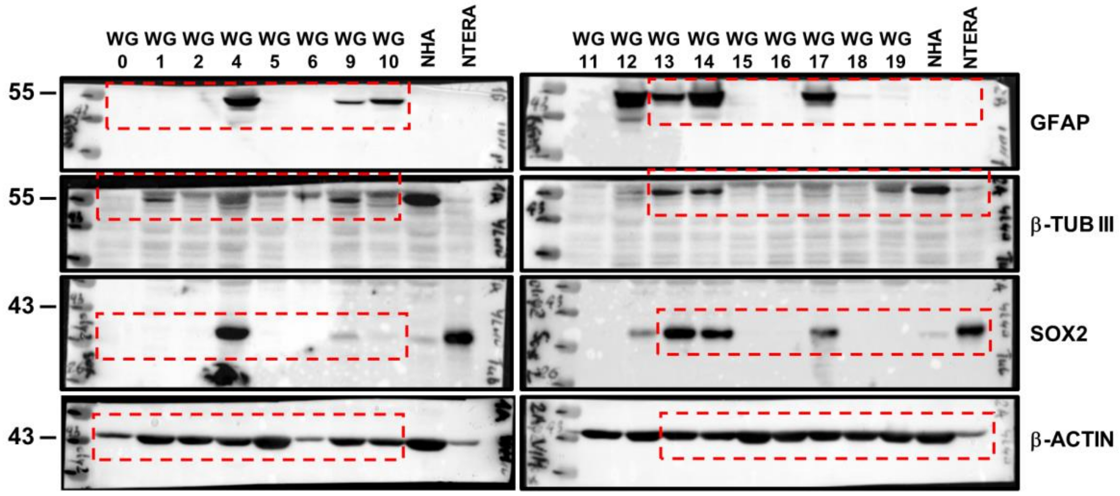

Figure S9. Western Blots related to Figure 2C.

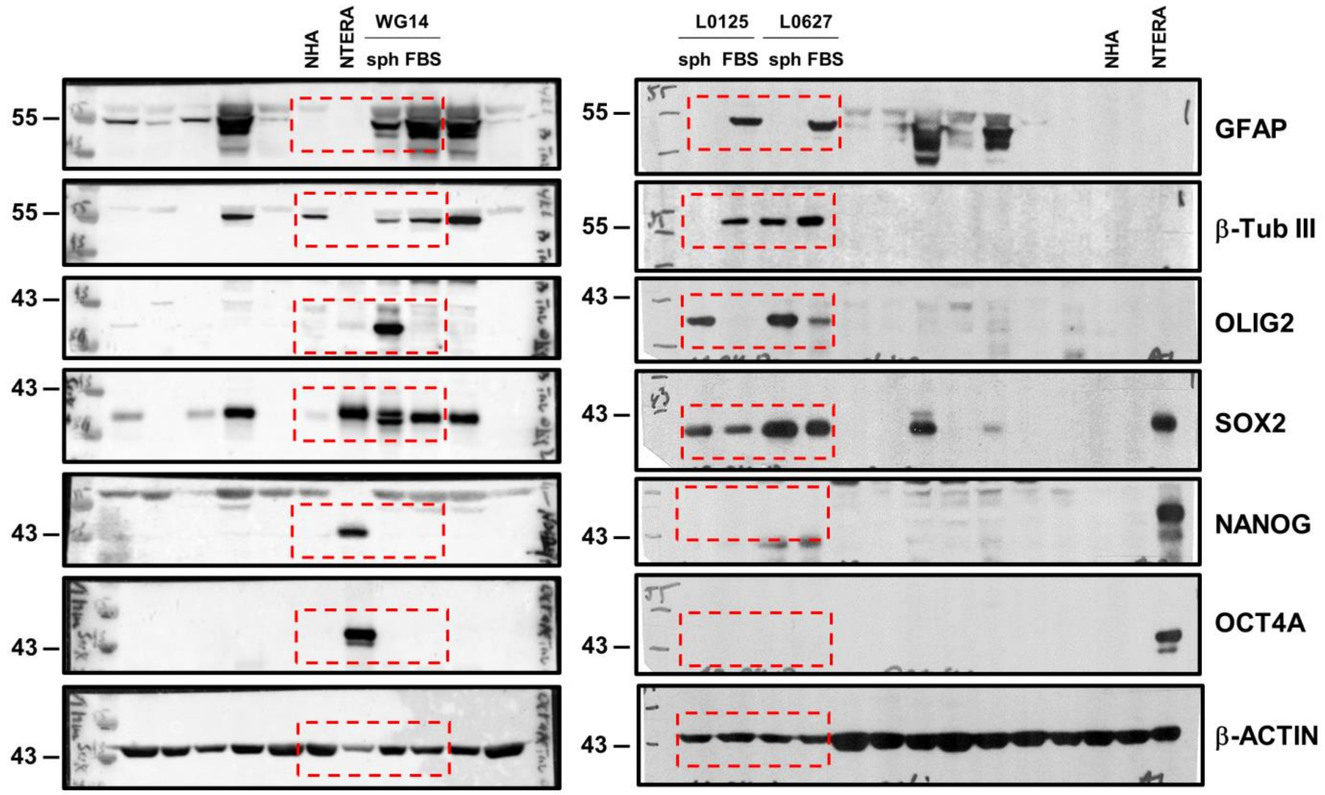

Figure S10. Western Blots related to Figure 3B.

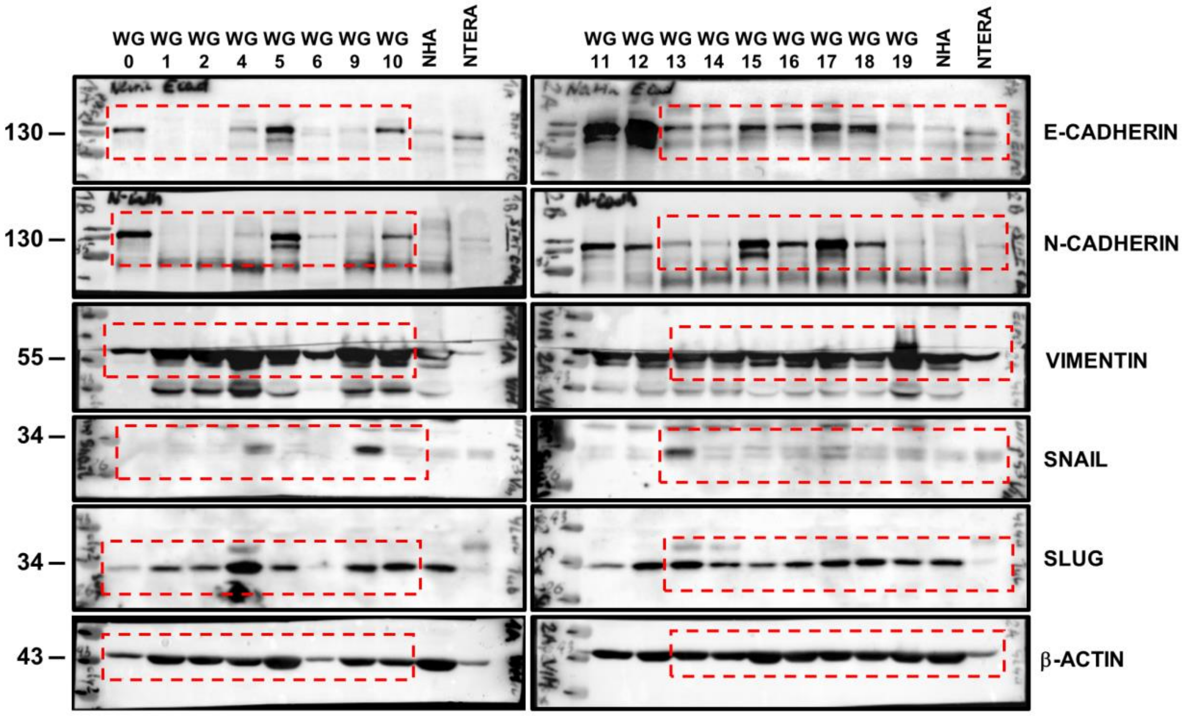

Figure S11. Western Blots related to Figure 4A.

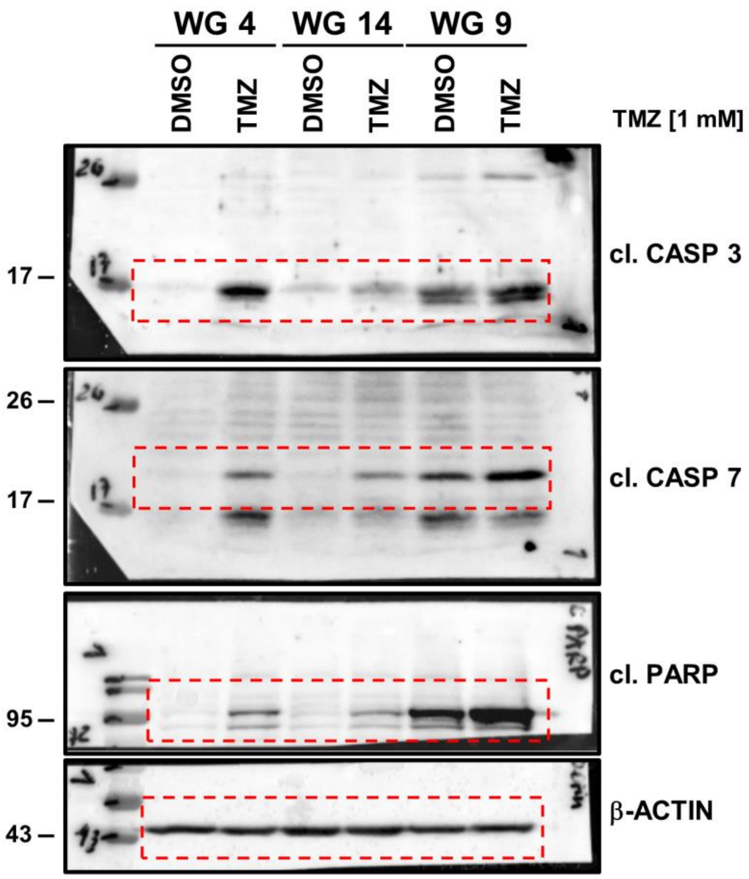

Figure S12. Western Blots related to Figure 5F.

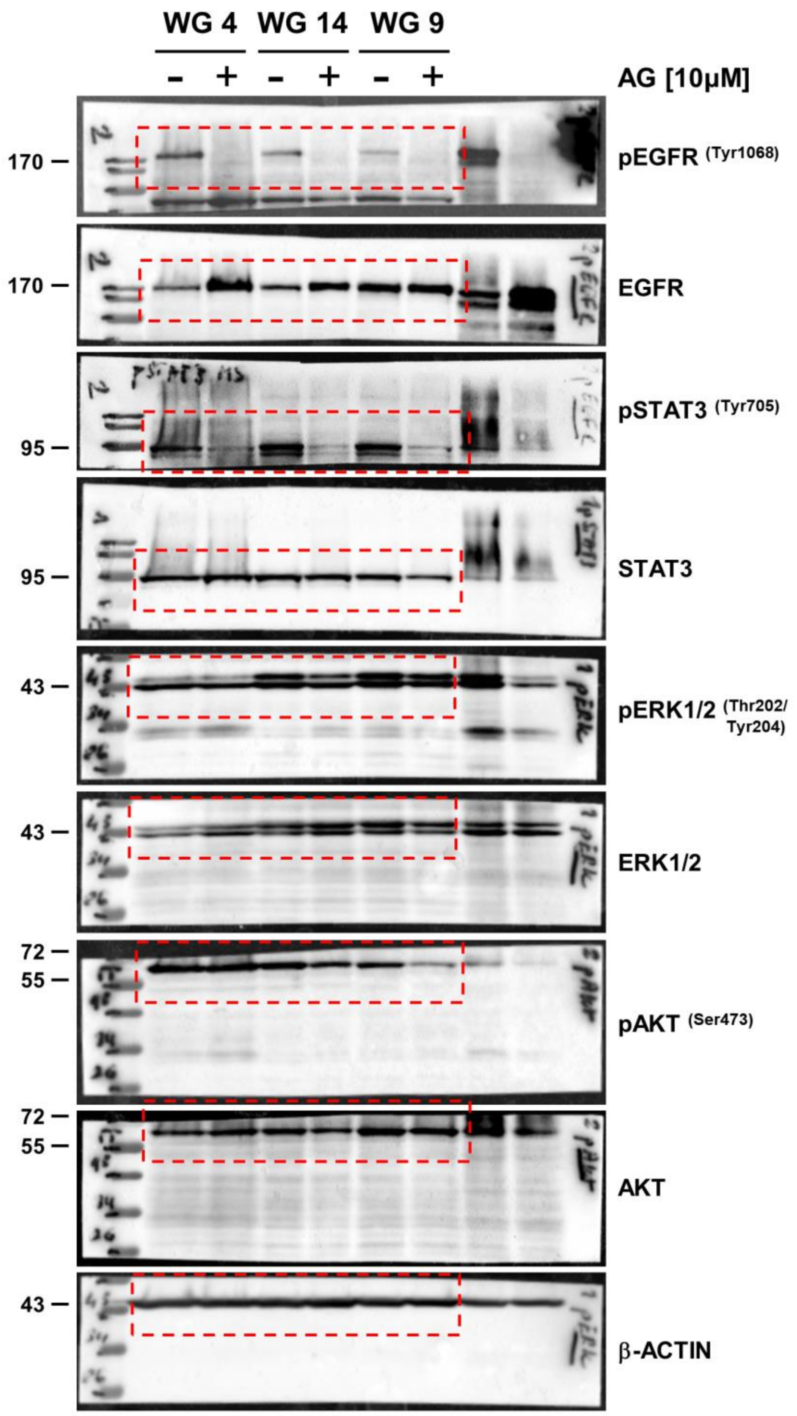

Figure S13. Western Blots related to Figure 6A.

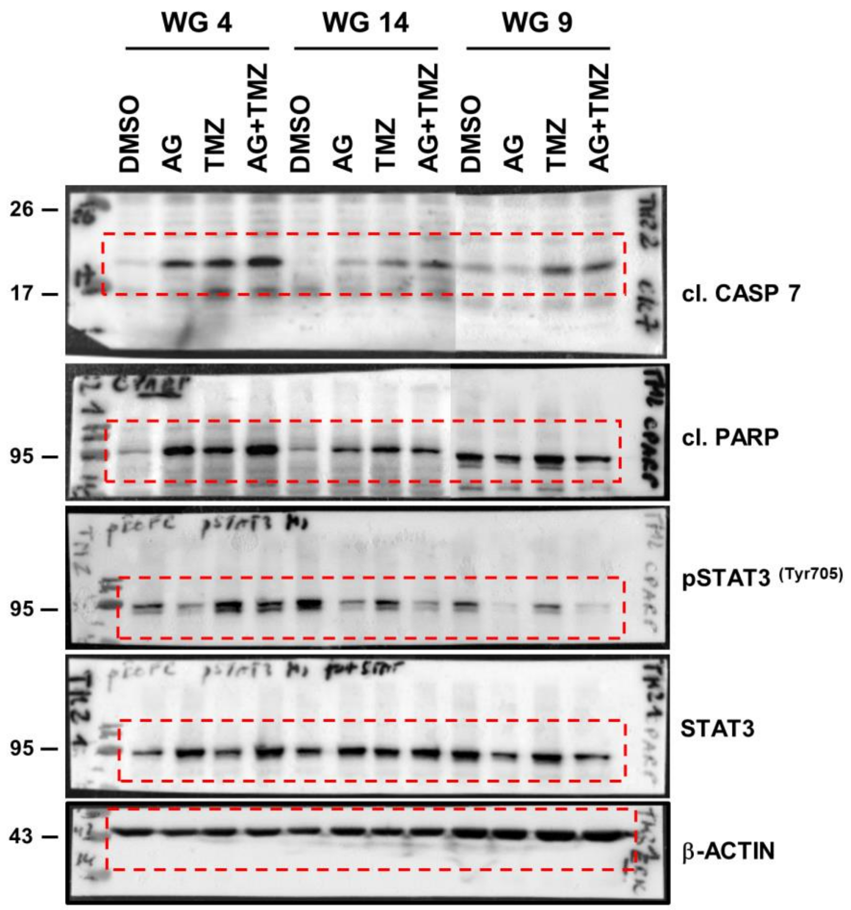

Figure S14. Western Blots related to Figure 6D.

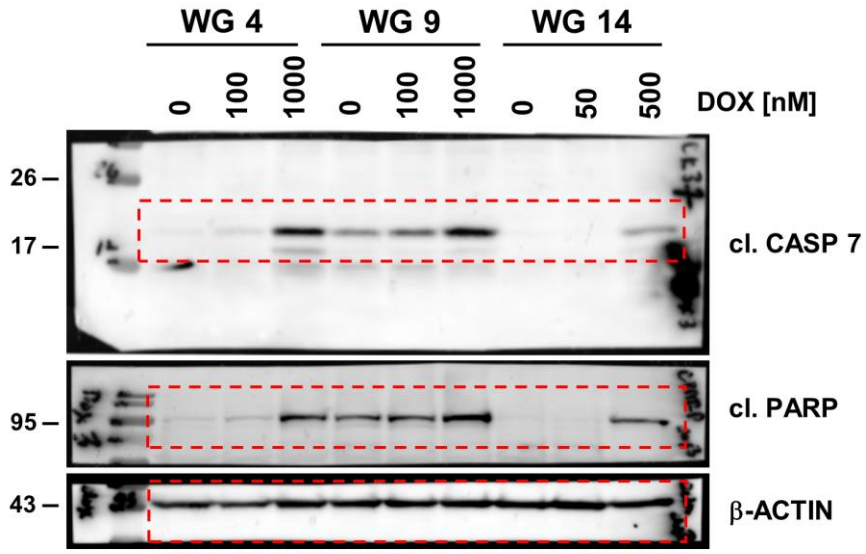

Figure S15. Western Blots related to Figure 7A.

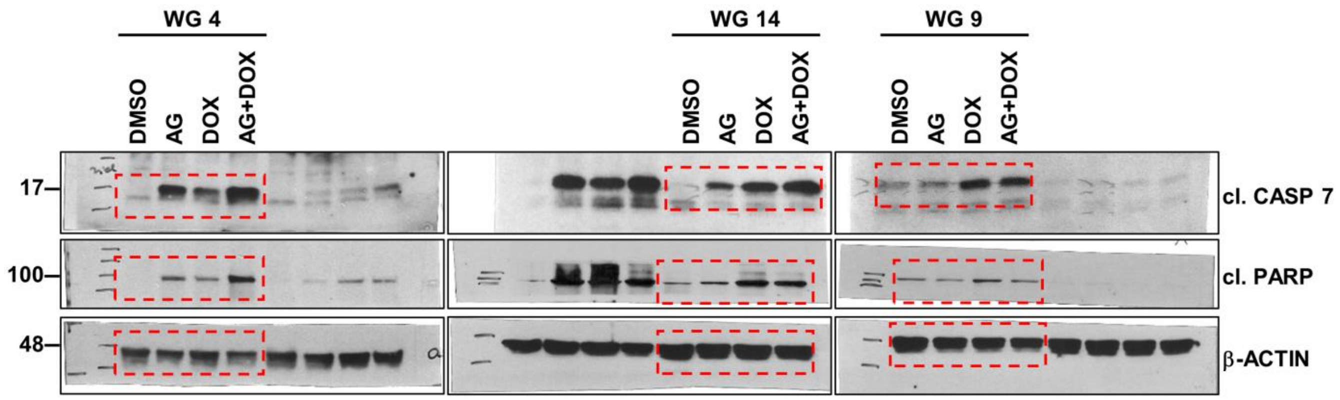

Figure S16. Western Blots related to Figure 7D.

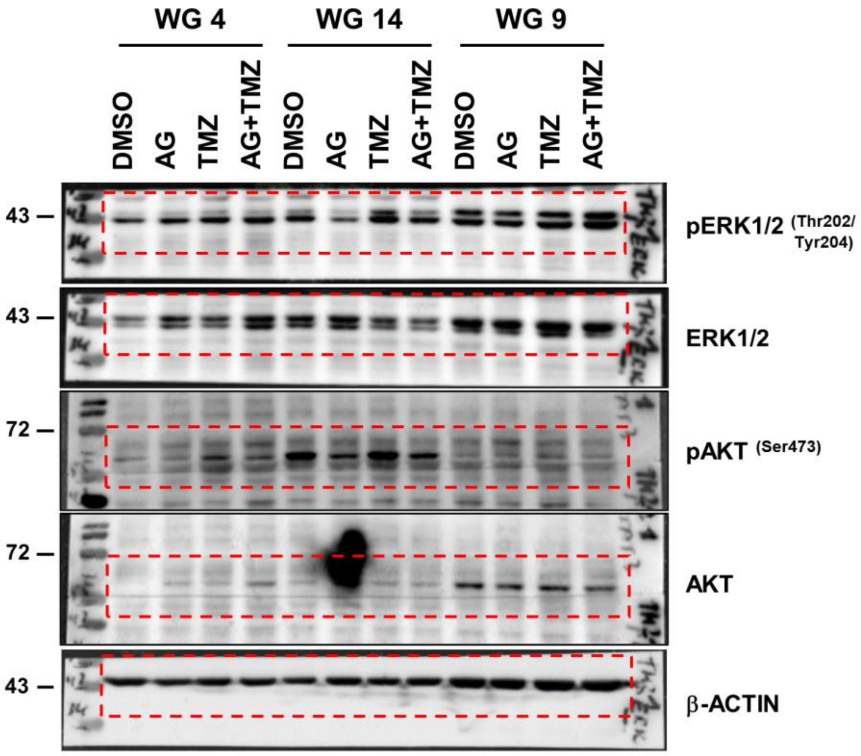

Figure S17. Western Blots related to Supplementary Figure S6D.

Table S1: Antibodies and reagents used.

Antibodies used for stainings				
**Antibody**	**Clone**	**Manufacturer**	**Cat. number**	**Dilution**
anti-GFAP Cocktail	4A11, 1B4, 2E1	BD Biosciences	555330	1:500
anti-TUBULIN beta III isoform	TU-20 (TUJ1)	Millipore	MAB1637	1:500
anti-NESTIN	196908	R&D Systems	MAB1259	1:100
anti-MAP2	AP20	STEMCELL Technologie		1:200
anti-OLIG2	2583426	Millipore	AB9610	1:500
anti-SOX2	D6D9	Cell Signaling	3579	1:300
Alexa Fluor 555 anti-rabbit	-	Invitrogen	A31572	1:1500
Alexa Fluor 555 anti-mouse	-	Invitrogen	A31570	1:1500
**Antibodies used for immunoblotting**				
**Antibody**	**Clone**	**Manufacturer**	**Cat. number**	**Dilution**
anti-AKT	-	Cell Signaling	9272	1:1000
anti-phospho-AKT (Ser473)	193H12	Cell Signaling	4058	1:1000
anti-E-CADHERIN	7H12	ThermoFisher	MA5-15711	1:1000
anti-N-CADHERIN	D4R1H	Cell Signaling	13116	1:1000
anti-cleaved Caspase3	-	Cell Signaling	9661	1:1000
anti-cleaved Caspase7	-	Cell Signaling	9491	1:1000
anti-cleaved PARP	-	Cell Signaling	9541	1:1000
anti-EGFR	D38B1	Cell Signaling	4267	1:1000
anti-phospho-EGFR (Tyr1068)	D7A5	Cell Signaling	3777	1:1000
anti-ERK1/2	-	Cell Signaling	9102	1:1000
anti-phospho-ERK1/2 (Thr202/Tyr204)	-	Cell Signaling	9101	1:1000
anti-NANOG	D73G4	Cell Signaling	4903	1:1000
anti-OCT4	C30A3	Cell Signaling	2840	1:1000
anti-SLUG	C19G7	Cell Signaling	9585	1:1000
anti-SNAIL	C15D3	Cell Signaling	3879	1:1000
anti-SOX2	D6D9	Cell Signaling	3579	1:1000
anti-STAT3	124H6	Cell Signaling	9139	1:1000
anti-phospho-STAT3(Tyr705)	M9C6	Cell Signaling	4113	1:1000
anti-VIMENTIN	D21H3	Cell Signaling	5741	1:1000
anti-OLIG2	2583426	Millipore	AB9610	1:4000
anti-TUBULIN beta III isoform	TU-20 (TUJ1)	Millipore	MAB1637	1:500
anti-GFAP Cocktail	4A11, 1B4, 2E1	BD Pharmingen	555330	1:800
anti-TP53	-	BD Pharmingen	610183	1:500
anti-CD44	-	R&D Systems	AF3660	1:1000
anti-CHI3L1	-	R&D Systems	AF2599	1:1000
anti-IDH1 R132H	H09	Dianova	DIA-H09	1:400
anti-PDGFRA/CD140a	-	ThermoFisher	PA5-17623	1:1000
horseradish peroxidase-conjugated monoclonal anti-β-actin	AC-15	Sigma Aldrich	A3854	1:30000
horseradish peroxidase-conjugated anti-rabbit IgG	-	Vector	PI-1000	1:5000
horseradish peroxidase-conjugated anti-mouse IgG	-	Vector	PI-2000	1:5000

Primers and reagents		
**Primer**	**Forward**	**Reverse**
GFAP	TCCTGGAACAGCAAAACAAG	CAGCCTCAGGTTGGTTTCAT
TUBB3	GTACGTGCCTCGAGCCATTCT	CGTGTAGTGACCCTTGGCCC
SOX2	GGGGAAAGTAGTTTGCTGCC	CGCCGCCGATGATTGTTATT

OLIG2	TCGCATCCAGATTTTCGGGT	AAAAGGTCATCGGGCTCTGG
NESTIN	CAAGACTTCCCTCAGCTTTCAG	AGGTGTCTCAAGGGTAGCAG
CDH1	CCCGCCTTATGATTCTCTGCTCGTG	TCCGTACATGTCAGCCAGCTTCTTG
CDH2	ATTTCCATCCTGCGCGTGAA	ATCAGCACAAGGATAAGCAGGA
VIM	GGCGAGGAGAGCAGGTTTC	TGGGTATCAACCAGAGGGAGT
CD44	CCATCTGTGCAGCAAACAACA	TTCAGGTGGAGCTGAAGCATT
18S	CGGACATCTAAGGGCATCAC	AACGAACGAGACTCTGGCAT
Methylated MGMT	TTTCGACGTTCGTAGGTTTTCGC	GCACTCTTCCGAAAACGAAACG
Unmethylated MGMT	TTTGTGTTTTGATGTTTGTAGGTTTTTGT	AACTCCACACTCTTCCAAAAACAAAACA
Spp1	QT01008798, Qiagen	
**Other reagents**		
**Chemical compounds**		**Source**
Temozolomide		Sigma-Aldrich
Doxorubicin hydrochloride		Sigma-Aldrich
AG1478		Calbiochem
**Software**		
GraphPad Prism 6.07		
BioRad Image Lab (ver. 5.2)		
